# Cysteine specific bioconjugation with benzyl isothiocyanates†

**DOI:** 10.1039/d0ra02934c

**Published:** 2020-04-16

**Authors:** László Petri, Péter A. Szijj, Ádám Kelemen, Tímea Imre, Ágnes Gömöry, Maximillian T. W. Lee, Krisztina Hegedűs, Péter Ábrányi-Balogh, Vijay Chudasama, György Miklós Keserű

**Affiliations:** Medicinal Chemistry Research Group, Research Centre for Natural Sciences Magyar tudósok krt 2 H-1117 Budapest Hungary keseru.gyorgy@ttk.hu; Department of Chemistry, University College London 20 Gordon Street London WC1H OAJ UK; MS Metabolomics Research Group, Research Centre for Natural Sciences Magyar tudósok krt 2 H-1117 Budapest Hungary; MS Proteomics Research Group, Research Centre for Natural Sciences Magyar tudósok krt 2 H-1117 Budapest Hungary; Department of Immunology, Eötvös Loránd University Pázmány Péter sétány 1/C H-1117 Budapest Hungary

## Abstract

Protein labelling has a wide variety of applications in medicinal chemistry and chemical biology. In addition to covalent inhibition, specific labelling of biomolecules with fluorescent dyes is important in both target discovery, validation and diagnostics. Our research was conducted through the fragment-based development of a new benzyl-isothiocyanate-activated fluorescent dye based on the fluorescein scaffold. This molecule was evaluated against fluorescein isothiocyanate, a prevalent labelling agent. The reactivity and selectivity of phenyl- and benzyl isothiocyanate were compared at different pHs, and their activity was tested on several protein targets. Finally, the clinically approved antibody trastuzumab (and it's Fab fragment) were specifically labelled through reaction with free cysteines reductively liberated from their interchain disulfide bonds. The newly developed benzyl-fluorescein isothiocyanate and its optimized labelling protocol stands to be a valuable addition to the tool kit of chemical biology.

## Abstract 2

We present herein the development of a new fluorescent dye equipped with a benzyl isothiocyanate warhead, which resulted improved photophysical properties and enhanced labelling efficiency on the Fab antibody subunit and the trastuzumab antibody.

## Graphical abstract

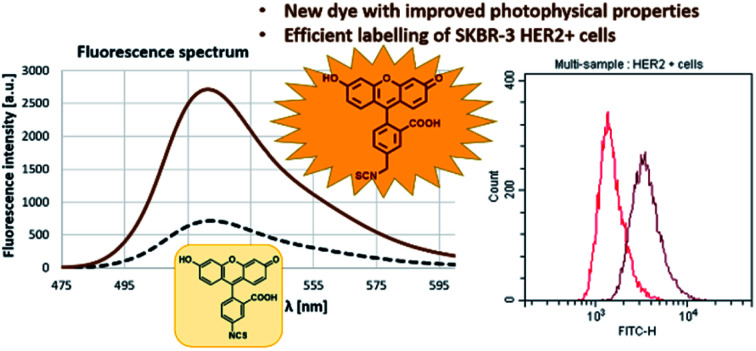

## Introduction

**Fig. 1:**
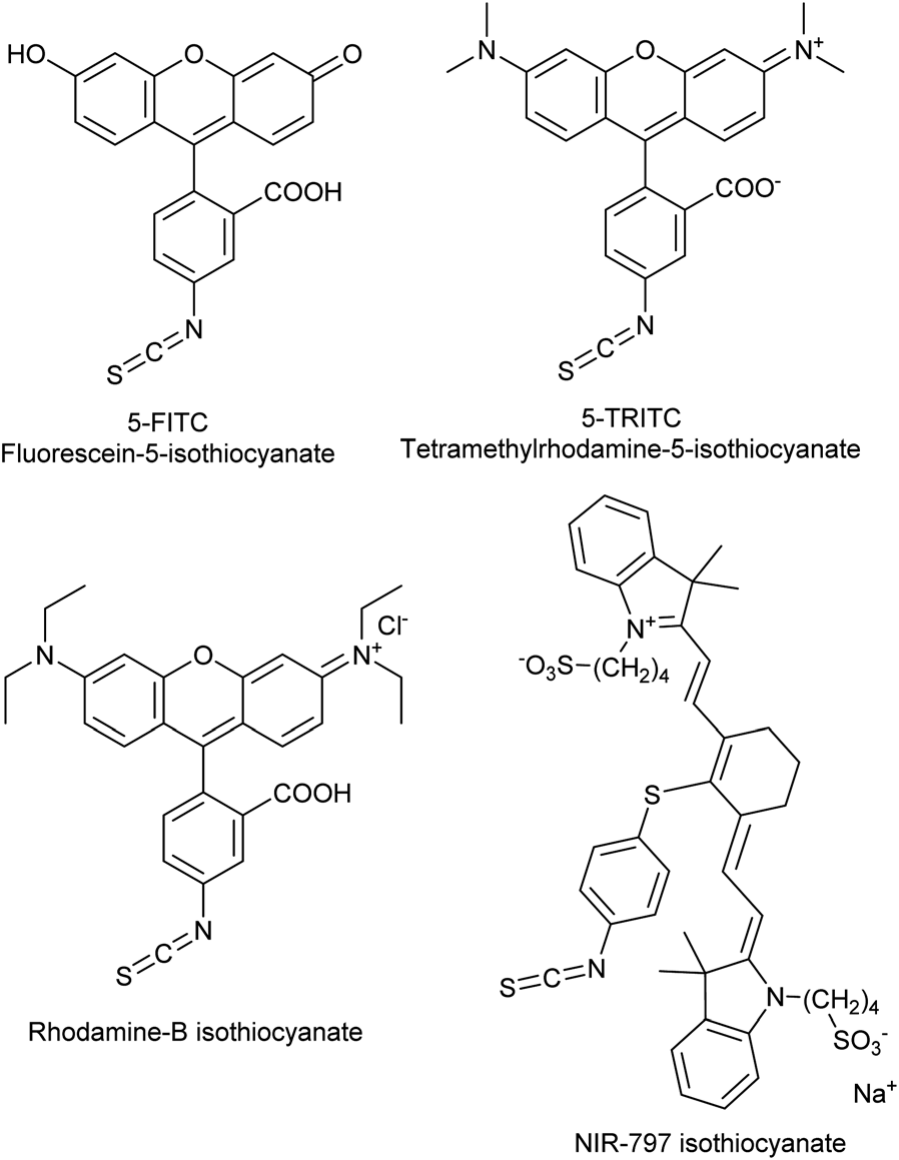
Frequently used dye-isothiocyanates.

The covalent labelling of proteins is a widespread approach in medicinal chemistry and chemical biology. In particular, developing irreversibly attached drugs, tagging biomolecules with fluorescent dyes for imaging and the design of antibody–drug conjugates are at the cutting edge of these fields.^1,2^ The formation of the covalent bond generally requires the presence of a nucleophilic amino acid residue in the protein and a small molecule equipped with an electrophilic centre. Usually cysteine and lysine are targeted, but in some cases tyrosine, threonine and serine might be modified, as well.^3^ In chemical biology, the dyes applied for direct labelling are often equipped with highly reactive maleimide, active ester, isothiocyanate or haloacetamide functional groups. Among other widely used isothiocyanates (ITCs, Fig. 1), fluorescein isothiocyanate (FITC) is a popular fluorescent labelling dye predominantly used for preparing a variety of fluorescent bioconjugates on lysines or cysteines.^4–6^ However, the low conjugation efficiency, the limited brightness and the short life time of its conjugates are still limiting applications.^7,8^

**Fig. 2:**
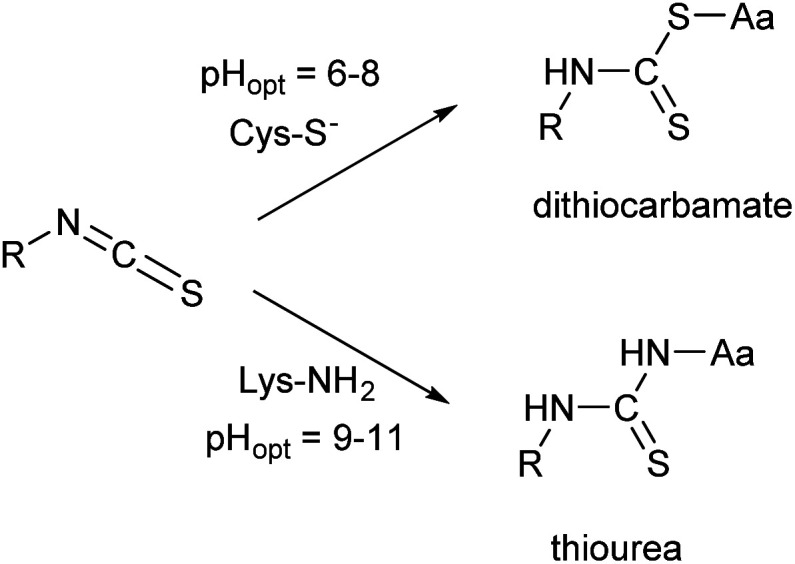
Reactivity of the isothiocyanate group with cysteine and lysine.

Many of these issues can be attributed to the characteristics of the isothiocyanate group. ITCs usually react with non-protonated aliphatic amine groups – including the terminal amines of proteins and the ε-amino groups of lysines – or with the thiolate form of cysteines (Fig. 2).^9,10^ The labelling selectivity between the amino acids targeted is mainly influenced by the pH of the surrounding media through the protonation state of the target amino acid side-chains. Amino groups are protonated at lower pH-values (NH_2_ → NH_3_^+^), thus lysine labelling by isothiocyanates may require pH 9.0–11.0 for optimal conjugation.^11^ Whereas, thiol reactivity is improved at weekly basic pH values (7.4–9.1)^12^ where lysines react slower. The labelling with ITCs is usually a very rapid reaction, but considering electronic effects, the electron-rich phenyl-isothiocyanate (PITC)-derivatives have lower reactivity, while EWG-substituted derivatives (*e.g.* FITC itself) show enhanced reactivity.^13,14^ One might see that in these cases the ITC group is conjugated to the electron system of the aromatic ring that might have a stabilizing, but reactivity-moderating effect. Notably, benzyl- (BITC), phenethyl- (PEITC) and various alkyl-substituted ITCs show significant reactivity as well.^15–18^

The labelling of antibodies with isothiocyanates has a long history of more than half a century and the application of FITC is still one of the most prevalent methods for the attachment of fluorophores to immunoglobulins.^19–28^ The goal of this research project was the systematic investigation of the pH-dependent reactivity and selectivity of ITCs and the development of a new, cysteine-selective fluorescein-based dye with enhanced labelling efficiency and improved conjugate-stability. The fluorescent probe was aimed to be applied for the labelling of the human, clinically approved, anti-HER2-antibody trastuzumab.

## Results and discussion

**Scheme 1:**
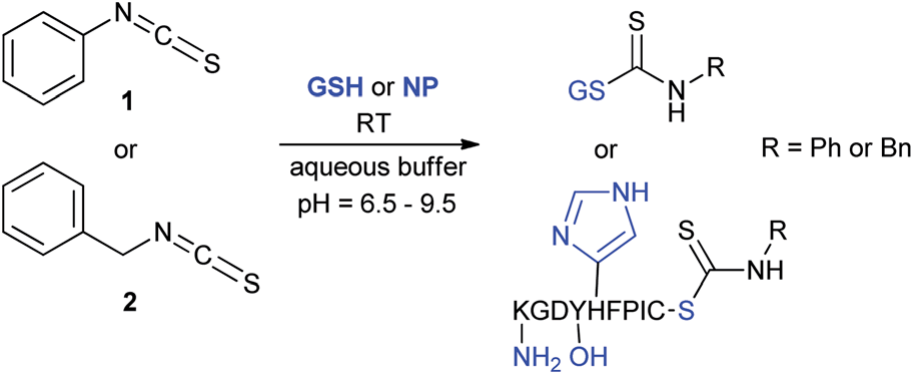
Reactivity of the isothiocyanate group with cysteine and lysine. For reactivity assay against GSH 20-times excess, for selectivity assay against NP 10-times excess was applied.

We have investigated the reactivity and selectivity of the isothiocyanate functional group depending on different pHs. The model compounds selected were phenyl isothiocyanate (1) and benzyl isothiocyanate (2) (Scheme 1). The reactivity of the two molecules was evaluated in a kinetic assay with l-glutathione (GSH) at four different pH values (6.5, 7.4, 8.0 and 9.5) in PBS buffer (Table 1).^29^ The amino acid selectivity was tested under the same conditions on a KGDYHFPIC nonapeptide (NP) containing Lys and Tyr nucleophilic residues besides Cys. The site of labelling was identified by HPLC-MS/MS measurements.

**Table 1:** pH-dependent reactivity and selectivity of phenyl isothiocyanate (1) and benzyl isothiocyanate (2) on surrogate models. For the reactivity assay 0.25 mM of fragments were screened in PBS buffer against 5 mM of GSH. For the selectivity assay 1 mM of fragments were incubated in PBS buffer together with 0.1 mM of NP for 16 h at 25 °C

Compound	pH	GSH 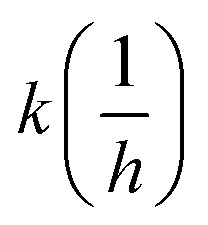	NP conversion and preferred amino acid
1	6.5	3.08	10% C
	7.4 (ref. 29)	4.25	10% C
	8.0	>13.9^a^	5% C
	9.5	>13.9	15% C
2	6.5	1.13	79% C
	7.4 (ref. 29)	2.12	73% C
	8.0	>13.9^a^	77% C, 5% C + K
	9.5	>13.9^a^	31% C, 24% C + K

a The reactions is faster than the minimal time window necessary to obtain LC-MS spectra are reported here with a kinetic rate constant > 13.9, due to the minimal running time is 3 min, which would be equal to 
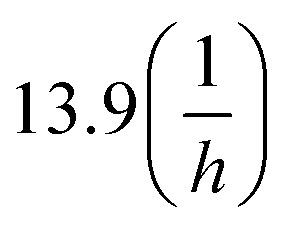
 kinetic rate constant.

The surrogate reactivity assay (Table 1, GSH) suggested considerable thiol-reactivity for both isothiocyanates, with reactivity increasing with pH. This can be rationalized with the larger amount of the thiolate form of Cys at higher pHs. The selectivity assay showed cysteine preference in all cases (Table 1, NP). It is noteworthy, that we can observe a trend in lysine labelling, as it is increasing in parallel with the pH. This is in line with the fact we previously described (Fig. 2) regarding the appropriate pH for the covalent labelling of cysteine and lysine.

Following the surrogate assays, the reactivity of the two isothiocyanates was screened on a set of pharmacologically relevant proteins possessing catalytic and non-catalytic cysteines. MurA expressed from *Escherichia coli* (MurA_EC_) or *Staphylococcus aureus* (MurA_SA_) are bacterial enzymes responsible for cell wall synthesis,^30^ while cathepsin B (with endo- and exopeptidase activity) and cathepsin X are human cysteine proteases.^31^ In addition, we investigated the intrinsically disordered tau, which has a significant effect in neurodegenerative disorders,^32^ and the oncogenic mutant KRas G12C.^33^ In the case of MurA_EC_, MurA_SA_, CatB_endo_, CatB_exo_ and CatX the biochemical assay results have been published previously as part of a larger screening campaign.^29^ The inhibition in the latter cases was quantified in a functional biochemical assay resulting in remaining activity values (RA%), while for the tau and KRas G12C targets we performed a high throughput thiol reactivity assay showing the remaining free thiol ratio (FTR%) after covalent labelling (Table 2).^34^ MurA and the cathepsins have catalytic cysteines, thus the inhibition measured is in direct relation with labelling. Low free thiol ratios measured for tau and KRas G12C would suggest high levels of cysteine labelling. The protein labelling results showed that both fragments were able to label different types of proteins, originating either from bacteria or human cells; influencing their activity. In addition, one might see that a wide range of labelling and inhibiting efficiency is covered, thus these ITCs shouldn't be considered as promiscuous agents. Notably, benzyl isothiocyanate (2) was found to be more effective in all cases tested, in some cases even being effective on proteins that were not labelled by ITC 1. A mentioned earlier, the labelling of catalytic cysteines is in direct relation with the inhibition. On the contrary, for tau and KRas G12C covalent binding to the targeted cysteines had to be proven. Therefore, we have confirmed the covalent labelling of tau by MS (Fig. S1†) and the modification of *Cys12* in KRas G12C by ^15^N-HSQC NMR (Fig. S2†). In details, with tau we identified double labelling on the available cysteines *Cys291* and *Cys322* with fragment 2, while labelling with isothiocyanate 1 showed only partial labelling. In the case of KRas G12C, based on the relative integrals of the NMR spectrum, we could observe significantly higher conversion with fragment 2 (54% with 1 and 98% with 2). These semiquantitative results obtained by MS and NMR analytics are in line with the results of the thiol reactivity assay. Altogether, after these protein labelling measurements, we consider the benzyl-isothiocyanate warhead a more effective one compared to its phenyl analogue.

**Table 2:** Protein labelling results of isothiocyanates 1 and 2 measured at 500 μM for MurAs and Cats and 200 μM for tau and KRas G12C

	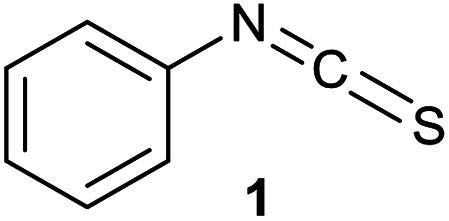	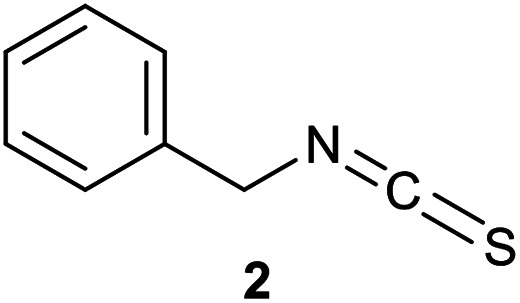
MurA_EC_ RA [%]^29^	53	3
MurA_SA_ RA [%]^29^	75	41
CatB^endo^ RA [%]^29^	100	75
CatB^exo^ RA [%]^29^	98	18
CatX RA [%]^29^	67	22
Tau FTR [%]	60	0
KRas G12C FTR [%]	62	14

Our next protein target to appraise was the antigen-binding fragment (Fab) of the anti-HER2 antibody trastuzumab, which was prepared by enzymatic digestion (ESI Fig. S3†). The interchain disulfide bridge of Fab was reduced with TCEP to liberate two free solvent-accessible cysteines. Subsequently, pH-dependent labelling assays were performed in PBS buffer at pH 6.5 and 8.0 with 1000-fold excess of each isothiocyanate. This high excess of reagent was employed to provide forcing conditions under which selectivity could be determined with confidence. It was postulated that if a molecule does not react with lysines under these stringent conditions, we can safely assume it is unreactive towards amine groups at the analysed pH values. The results were analysed by HPLC-MS (Table 3, ESI Fig. S4–S7†).

**Table 3:** pH-dependent modification of reduced trastuzumab Fab by isothiocyanates 1 and 2 measured at 10 mM concentration. Incubation was conducted with 1000-fold excess of the fragment at 37 °C for 90 min^a^

Compound	Reduced Fab	
	pH = 6.5	pH = 8.0
1	No modification^b^	11% single mod. on LC
		13% single mod. on HC
2	100% single mod. on LC	100% single mod. on LC
	100% single mod. on HC	100% single mod. on HC

a LC and HC refer to the light chain and heavy chain of the Fab and yield of labelling was determined for the observed labelled/unlabelled LC/HC agents, respectively.

b In this experiment a significant amount of reconjugated Fab was observed. However, full reduction was confirmed before, suggesting that only partial bioconjugation occured.

Notably, while evaluating the MS spectra, sometimes adduct ions with 98 Da mass difference were observed corresponding to adducts with inorganic ions (*e.g.* phosphate, sulfate), which often appear during ESI ionization of protein samples.^35^ Phenyl isothiocyanate (1) showed no significant labelling under any conditions applied. On the contrary, benzyl isothiocyanate labelled the reduced Fab completely both at pH = 6.5 and 8.0. Additionally, in order to assess whether any lysine modification occurred, benzyl isothiocyanate (2) was incubated with native Fab. We obtained no labelling at pH = 6.5 or 8.0, both (ESI Fig. S8 and S9,† respectively), suggesting that the strong reactivity with reduced Fab is derived from the selective labelling of cysteines.

**Scheme 2:**
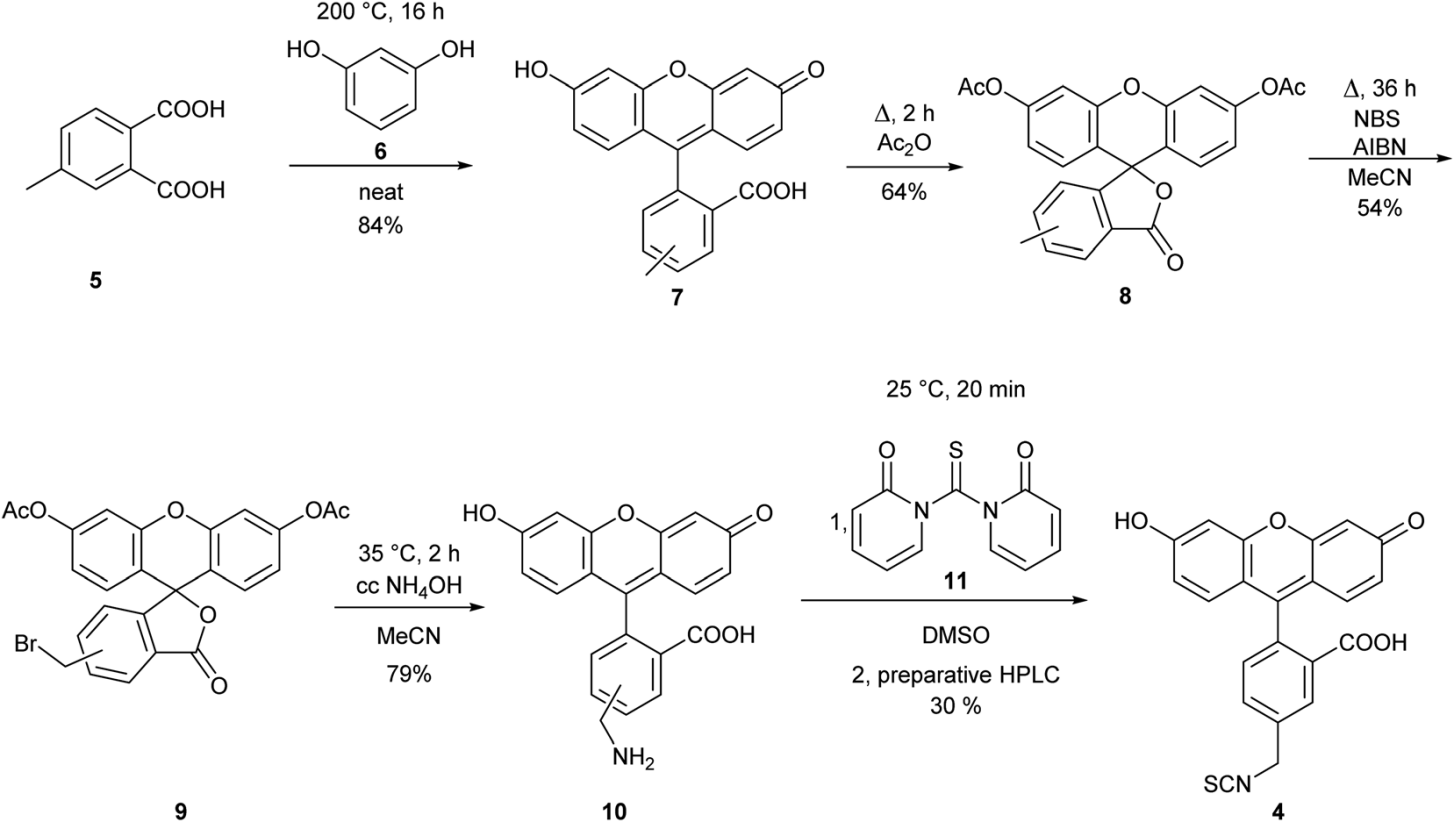
Synthesis of FBITC (4).

The observed differences in the reactivity of isothiocyanates 1 and 2 suggested that modifying a phenyl isothiocyanate type fluorescent dye to the corresponding benzyl isothiocyanate would improve the labelling efficiency. Thus, we selected fluorescein isothiocyanate (FITC, 3), a commonly used fluorescent labelling agent, and designed its benzylic analogue (FBITC, 4). To this end, resorcinol (5) and 4-methyl-phthalic acid (6) were heated together at 200 °C overnight, generating the methyl-fluorescein core in good yield (84%). Two regioisomers were produced, as the methyl group of the phthalic acid could end up in positions 4 or 5. These were not separated at this stage. Following an acetylation of the phenolic OH groups with refluxing acetic anhydride, the methyl group was brominated at room temperature, in order to keep the selectivity for the mono-bromination, by NBS in the presence of AIBN in a moderate yield (54%). The removal of the acetyl groups and the substitution of the bromine to an amino function was accomplished smoothly in one step using conc. aq. ammonia at 35 °C. Finally, the amine was converted to isothiocyanate by reacting 10 with 1,1′-thiocarbonyldi-2(1*H*)-pyridone (11), and the regioisomers were separated by preparative HPLC resulting in FBITC 4 (Scheme 2).

**Fig. 3:**
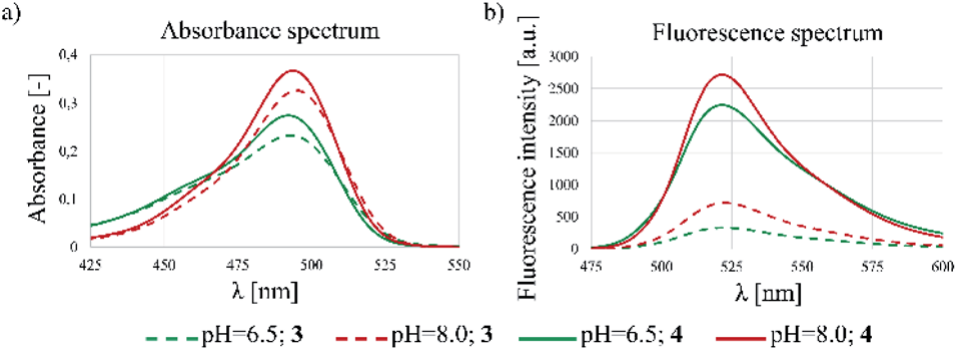
Fluorescent spectrometry results of 3 and 4 dyes. (a) Absorbance and (b) fluorescence spectra at different pHs (6.5 and 8.0).

The spectrophotometric properties of FBITC (4) were compared to that of FITC (3) investigating them at different pH-values (PBS buffer, 10% DMSO, pH = 6.5 and 8.0) (Fig. 3). Notably, both compounds showed larger absorbance at pH = 8.0 with only a slight difference between 3 and 4. More importantly, however, a significant improvement in fluorescence intensity was observed for compound 4 at both pH values. This finding has been confirmed by the comparative analysis of molar absorption coefficients, quantum yields and emitted brightness (Table 4).

**Table 4:** Spectrophotometric properties of 3 and 4

	3		4	
pH	6.5	8.0	6.5	8.0
*λ* ^max^_abs_ [nm]	495	495	493	494
*λ* ^max^_em_ [nm]	523	522	521	522
Molar absorption coefficient @ *λ*^max^_abs_ [M^−1^ cm^−1^]	43 107	67 302	56 970	73 519
Quantum yield	0.053	0.16	0.35	0.56
Brightness	2304	10 874	20 164	40 946

The evaluation of FBITC (4) suggested that this new dye might show improved efficiency in labelling proteins compared to FITC (3). Thus, the pH-dependent reactivity of dyes 3 and 4 was tested on the Fab region of trastuzumab (Table 4, ESI Fig. S10–S17†). Labelling of the reduced Fab was performed in PBS buffer at pH = 6.5 and pH = 8.0 applying a lower, ten-fold excess of the isothiocyanates as these larger dyes are more expensive to manufacture than the fragment-sized ITC molecules tested before (1 and 2). Gratifyingly, the reaction proceeded well under these more financially viable conditions. FITC (3) labelled the reduced Fab moderately at both pH values and showed slight labelling on the native Fab at pH = 8.0. On the contrary, FBITC (4) reacted with high efficacy with the reduced Fab at both pHs and showed cysteine selective modification as no or minimal labelling of native (non-reduced) Fab was observed at pH 6.5 and 8.0, respectively (Table 5).

**Table 5:** pH-dependent modification of reduced trastuzumab Fab by isothiocyanates 3 and 4. Incubation was conducted with tenfold excess of the dye at 37 °C for 90 min^a^

	Reduced Fab		Native Fab	
	pH = 6.5	pH = 8.0	pH = 6.5	pH = 8.0
3	LC: 41%, single mod.	LC: 57%, single mod.^b^	No mod.	21% single mod.
	HC: 36%, single mod.	HC: 64%, single mod.^b^		
4	LC: 89%, single mod.	LC: 100%, single mod.	No mod.	8% single mod.
	HC: 100%, single mod.	HC: 100%, single mod.		

a LC and HC refer to the light chain and heavy chain of the Fab and yield of labelling was determined for the observed labelled/unlabelled LC/HC agents, respectively.

b In this experiment a significant amount of reconjugated Fab was observed. However, full reduction was confirmed before, suggesting that only partial bioconjugation occured.

**Fig. 4:**
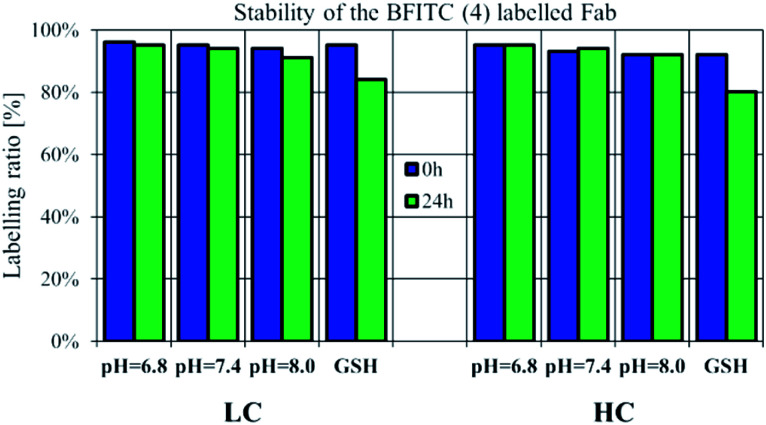
Stability of 4-labelled reduced Fab measured by LC-MS/MS in different pH conditions. Labelled LC/HC was incubated in acidic (pH = 6.8), physiologic (pH = 7.4) and basic (pH = 8.0) conditions and additionally at pH = 7.4 with GSH (5 μM).

Checking the suitability of FBITC (4) as an irreversible labelling agent, the stability of its Fab conjugate was tested by HPLC-MS over a time range of 24 h. The conjugation was performed at pH = 8.0, followed by buffer exchange of the construct to pH = 7.4 PBS. Stability measurements were performed with acidic and basic pH-shift, as well, to test pH-dependent cleavability of the dye 4 labelled LC/HC. In addition, we also performed stability measurements of the labelled Fab chains in the presence of 5 μM GSH.^36^ The samples were kept at room temperature and in all cases no significant change in the labelling ratio was observed after 24 h (Fig. 4, S18–S21†). Here we report the labelling ratios, which were calculated by the deconvoluted spectra peak intensity of the labelled LC/HC in proportion to the cumulative intensity of labelled and unlabelled LC/HC. Thus, we concluded that pH-shift and thiol access had no effect on the stability of the labelled LC/HC.

**Fig. 5:**
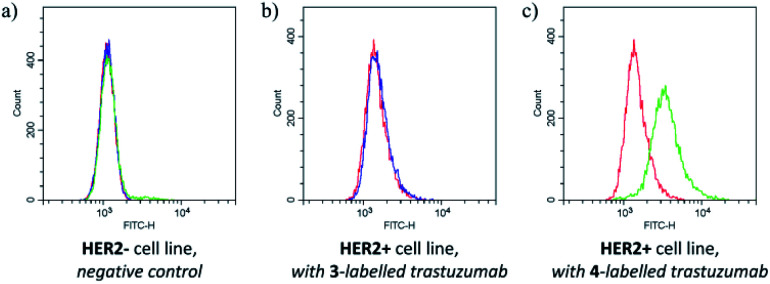
Results of flow cytometry measurements on (a) HER2− and (b) HER2+ cells with 3- and (c) with 4-labelled trastuzumab. The colours are as follows: red for negative control to test autofluorescence, blue for FITC (3) dye labelled trastuzumab and green for FBITC (4) labelled trastuzumab.

The labelling efficiency and the stability of the conjugate suggested that FBITC (4) might be a useful tool for labelling trastuzumab full antibody. Considering that reduced Fab has two free cysteines, but the full antibody has eight in its reduced form, the excess of the dyes was increased to 40 equivalents for this experiment. The conjugation was completed in PBS buffer at pH = 8.0 with both FITC (3) and FBITC (4). Efficient labelling was confirmed by LC-MS (Fig. S23†), then the preserved biological function of trastuzumab was tested by flow cytometry on HER2+ cells (Fig. 5). Treating SKBR-3 cells with FITC (3) labelled antibody did not cause noteworthy change (median shift: 1500.4) compared to the autofluorescence (median shift: 1428.4), while the fluorescent signal for the 4-labelled antibody shifted significantly suggesting better fluorescent labelling (median shift: 3404.8). One could conclude that the bioconjugation preserves the biological function of the antibody and that FBITC (4) is a significantly better labelling reagent than FITC (3) under these conditions.

Even though FITC is a commonly used reagent for lysine labelling of proteins, we envision that cysteine labelling may be advantageous in some cases. Lysine labelling will always result in a heterogeneous mixture of conjugates due to the large number of surface accessible lysines on antibody residues, and this reduces reproducibility and batch-to-batch consistency. In contrast, on a reduced antibody the maximum number of eligible cysteines is 8, and thus full modification to produce a homogeneous product is feasible. But even with a fluorophore-antibody ratio (FAR) of lower than 8, the product will be far less heterogeneous due to the much lower number of possible attachment sites (8 as opposed to ∼50). Furthermore, as lysine modification is usually carried out at higher pHs of 9–11, and indeed, our data shows that minimal lysine-modification of trastuzumab by FITC was observed at pH 8, for base-sensitive proteins the milder cysteine-selective protocol reported here would be advantageous. It is also important to note that these findings of the benzylic analogue of FITC increasing cysteine-reactivity and fluorophore brightness may also hold true for other fluorescent dyes that are based on the fluorescein platform such as AlexaFluors. These dyes are normally sold as their NHS-ester activated variants for lysine modification, but it would be interesting to compare their properties to their benzylic ITC variants.

## Experimental

### Instruments and methods

^1^H NMR spectra were recorded in DMSO-*d*_6_ or CDCl_3_ solution at room temperature, on a Varian Unity Inova 500 spectrometer (500 MHz for ^1^H NMR spectra), with the deuterium signal of the solvent as the lock and TMS as the internal standard. Chemical shifts (*δ*) and coupling constants (*J*) are given in ppm and Hz, respectively.

HPLC-MS measurements were performed using a Shimadzu LCMS-2020 device equipped with a Reprospher 100 C18 (5 μm; 100 × 3 mm) column and positive-negative double ion source (DUIS±) with a quadrupole MS analyzer in a range of 50–1000 *m*/*z*. Sample was eluted with gradient elution using eluent A (10 mM ammonium formate in water : acetonitrile 19 : 1) and eluent B (10 mM ammonium formate in water : acetonitrile 1 : 4). Flow rate was set to 1 mL min^−1^. The initial condition was 0% B eluent, followed by a linear gradient to 100% B eluent by 1 min, from 1 to 3.5 min 100% B eluent was retained; and from 3.5 to 4.5 min back to initial condition with 5% B eluent and retained to 5 min. The column temperature was kept at room temperature and the injection volume was 10 μL. Purity of compounds was assessed by HPLC with UV detection at 215 nm; all tested compounds were >95% pure.

The molecular weights of the conjugates of trastuzumab Fab were identified using a Triple TOF 5600+ hybrid Quadrupole-TOF LC/MS/MS system (Sciex, Singapore, Woodlands) equipped with a DuoSpray IonSource coupled with a Shimadzu Prominence LC20 UFLC (Shimadzu, Japan) system consisting of binary pump, an autosampler and a thermostated column compartment. Data acquisition and processing were performed using Analyst TF software version 1.7.1 (AB Sciex Instruments, CA, USA). Chromatographic separation was achieved on a Thermo Beta Basic C8 (50 mm × 2.1 mm, 3 μm, 150 Å) HPLC column. Sample was eluted in gradient elution mode using solvent A (0.1% formic acid in water) and solvent B (0.1% formic acid in ACN). The initial condition was 20% B for 1 min, followed by a linear gradient to 90% B by 4 min, from 5 to 6 min 90% B was retained; and from 6 to 6.5 min back to initial condition with 20% eluent B and retained from 6.5 to 9.0 min. Flow rate was set to 0.4 mL min^−1^. The column temperature was 40 °C and the injection volume was 5 μL. Nitrogen was used as the nebulizer gas (GS1), heater gas (GS2), and curtain gas with the optimum values set at 30, 30 and 35 (arbitrary units), respectively. Data were acquired in positive electrospray mode in the mass range of *m*/*z* = 300 to 2500, with 1 s accumulation time. The source temperature was 350 °C and the spray voltage was set to 5500 V. Declustering potential value was set to 80 V. Peak View Software™ V.2.2 (version 2.2, Sciex, Redwood City, CA, USA) was used for deconvoluting the raw electrospray data to obtain the neutral molecular masses. The MS evaluations were as follows, about 1.5 min chromatographic time was averaged in the LC run after background subtraction resulting in a mixed envelope both of the modified and unmodified light chain and the heavy chain of Fab, as well. The neutral molecular weights of the components can be obtained after deconvolution the raw spectrum. The labelling ratios are defined in % and calculated as the height ratios of the labelled and un-labelled peaks in the deconvoluted spectrum in case of heavy and the light chain related to the sum of the height intensities of the labelled and un-labelled species, respectively.

SKBR-3 cells were available from the American Type Culture Collection (ATCC) and maintained according to their specifications. For flow cytometric experiments 1–3 × 10^5^ cells per sample were harvested by trypsinization, washed in PBS containing 1% FCS. Cells were incubated in the presence of 1 μg labelled antibody for 30 min at 40 °C, and then measured with a Cytoflex (Beckman Coulter) flow cytometer using CytExpert 2.2 software for the evaluation of the results.

### Elmann's assay analysis of tau and KRas G12C

To measure thiol-reactivity, 2 μM of the target (tau and KRas-G12C) in assay buffer (25 mM NaH_2_PO_4_, 0.1 mM EDTA, 150 mM NaCl, pH = 6.6) was treated with 200 μM of fragments, resulting 5% DMSO concentration in the mixture. After 2 hours of incubation on room temperature, 16 μL of the sample was pipetted into a black, 384 well assay plate (Corning, Ref No.: 4514) and 4 μL of thiol detection reagent (Invitrogen, Ref No.: TC012-1EA) was added. After brief shaking, the plate was incubated in dark, room temperature for 30 min, then fluorescence was measured in duplicates in a microplate reader (BioTek Synergy Mx) (*λ*_ex_ = 390 nm and *λ*_em_ = 510 nm). Free thiol ratio (FTR%) were calculated, as follows:
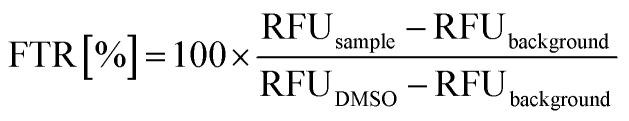


### Protocol for tau protein labelling

For the tau labelling experiment 25 μL of 10 μM stock solution of tau-K18 in 25 mM NaH_2_PO_4_ buffer at pH 6.6 with 150 mM NaCl and 0.1 mM EDTA was threated with 0.25 μL of 100 mM DMSO stock solution of the fragments. The mixture was then incubated at room temperature for 12 h. After the labelling, the mixture was subjected for MS analysis.

### ^15^N-HSQC NMR analysis of KRas G12C

NMR measurements for testing binding fragments to KRas4B-G12C-GDP protein were carried out on a Bruker Avance III 700 MHz spectrometer equipped with a 5 mm Prodigy TCI H&F-C/N-D, z-gradient probehead operating at 700.05 MHz for ^1^H and 70.94 MHz for ^15^N. Spectra were recorded at 298 K. For NMR samples the compounds were dissolved in DMSO in 20 mM concentration. To obtain reference spectra for the protein were measured in ^15^N-labeled KRAS4B-G12C1-169 (catalytic domain) mutant in 0.2 mM concentration, 5 mM GDP, 10 mM EDTA, 15 mM MgCl_2_ in PBS buffer (pH 7.4), 10% D_2_O, 5% DMSO and 1% DSS standard. NMR samples for binding tests contained ^15^N-labeled KRas4B-G12C in 0.2 mM concentration, 5% fragment stock solution (in DMSO, the final concentration of the compound is 1 mM), 2–3 mM GDP, 3–5 mM EDTA, 8–10 mM MgCl_2_ in PBS buffer (pH 7.4) and 10% D_2_O.

In binding tests 2D ^1^H, ^15^N-SOFAST-HMQC (NS = 64) spectra were performed subsequently immediately after mixing (*i.e.* <1 h) and after 1 day incubation (24 h) at room temperature. Sequence specific assignment of HN and N in the bound KRas4B-G12C spectra were transferred from previous results.^37^ All ^1^H chemical shifts were referenced to the DMSO peaks (which were calibrated to DSS resonance before in free protein measurements) as DSS were not added to avoid any side reactions with the limited amount of small molecules. ^15^N chemical shift values were referenced indirectly using the corresponding gyromagnetic ratios according to IUPAC convention. All spectra were processed with Bruker TOPSPIN. Binding was confirmed in every case by SOFAST-HMQC spectra: based on the compound evidenced by comparing SOFAST-HMQC spectra of free KRas-G12C-GDP and isothiocyanate-binded protein. Based on the assignment of SOFAST-HMQC spectra KRas-G12C-GDP, the cysteins modified covalently by the fragments were determined as well.

### Fragment reactivity and selectivity assays

The GSH-reactivity and nonapeptide-selectivity assays were reproduced as published in ref. 29, applying the appropriate pH-value.

### Preparation of trastuzumab Fab

Trastuzumab was purchased from UCLH in its clinical formulation. Trastuzumab Fab was prepared by a sequential enzymatic digest of the full antibody with pepsin and papain, following a literature procedure.^38^

### Procedure for reduction of trastuzumab Fab

A solution of 20 mM TCEP was prepared by dissolving TCEP (5.6 mg) in water (977 μL). Fab solution (20 μM, 250–500 μL) was prepared in (PBS 37 mM, pH 7.4) and 5 equivalents of TCEP (1 μL/200 μL of Fab solution) added. The mixture was incubated for 90 min at 37 °C. The resultant reduced Fab solution was buffer exchanged into the desired buffer (PBS 37 mM, pH 6.5 or 8.0).

### Procedure for bioconjugation reaction of trastuzumab Fab

A stock solution of the labelling molecule was prepared by dissolving 0.1 mmol of compounds 1–4 in DMSO (1000 μL). The resultant 100 mM solution was diluted to 20 mM using the desired buffer (PBS 37 mM, pH 6.5 or 8.0). To aliquots of the reduced Fab solution (20 μM, 20 μL) in the desired buffer (PBS 37 mM, pH 6.5 or 8.0) a solution of compounds 1–4 was added (20 mM, 20 μL for 1000 equivalents or 2 mM, 2 μL for 10 equivalents), and the reaction incubated at 37 °C. Samples were taken after 90 min and submitted for LC-MS analysis.

### Reduction and conjugation of trastuzumab

To a solution of trastuzumab (100 μL, 20 μM) in PBS buffer (37 mM PBS, 137 mM NaCl, 2.7 mM KCl, 2.0 mM EDTA, pH = 8.0) was added TCEP·HCl solution (10 equiv., 1 μL, 20 mM). The reaction mixture was incubated at 37 °C for 1.5 h. After this time, the solution was buffer exchanged into PBS buffer with Zeba™ Spin Desalting Columns (7k MWCO, Thermo Scientific™) to remove excess TCEP. Afterwards a DMSO solution of FBITC (4, 40 equiv., 14.68 μL, 5.45 mM) or of FITC (3, 40 equiv., 14.68 μL, 5.45 mM) was added to the reaction mixture, and it was incubated for 20 hours at room temperature. The samples were desalted (Zeba™ Spin) and analysed by LC-MS.

### Synthetic procedures

#### 2-(6-Hydroxy-3-oxo-3*H*-xanthen-9-yl)-4/5-methylbenzoic acid (7)

The mixture of 4-methylphthalic acid (2.0 g, 11.12 mmol) and resorcinol (2.45 g, 22.24 mmol, 2 equiv.) was heated at 200 °C overnight in a flask equipped with reflux condenser. The reaction was cooled down and the crude product was dissolved in 3 M HCl at 100 °C. The precipitated product was filtered and washed with water resulting in the mixture of 2-(6-hydroxy-3-oxo-3*H*-xanthen-9-yl)-4-methylbenzoic acid and 2-(6-hydroxy-3-oxo-3H-xanthen-9-yl)-5-methylbenzoic acid (3.23 g, 9.31 mmol, 84%). ^1^H NMR (500 MHz, CDCl_3_-MeOD) *δ* 7.86 (d, *J* = 7.9 Hz, 1H), 7.77 (s, 1H), 7.49 (d, *J* = 7.9 Hz, 1H), 7.40 (d, *J* = 7.9 Hz, 1H), 7.34 (s, 1H), 7.06 (d, *J* = 7.8 Hz, 1H), 7.00 (t, *J* = 8.0 Hz, 1H), 6.95 (s, 1H), 6.69 (d, *J* = 2.3 Hz, 1H), 6.61 (d, *J* = 2.8 Hz, 1H), 6.59 (d, *J* = 2.8 Hz, 1H), 6.53 (d, *J* = 2.4 Hz, 1H), 6.52 (d, *J* = 2.5 Hz, 1H), 6.52 (d, *J* = 2.5 Hz, 1H), 6.51 (d, *J* = 2.4 Hz, 1H), 6.34 (d, *J* = 2.3 Hz, 1H), 6.33 (d, *J* = 2.3 Hz, 1H), 6.31 (t, *J* = 2.2 Hz, 1H), 2.50 (s, 1H), 2.39 (s, 1H) ppm, APT NMR (125 MHz, CDCl_3_-MeOD) *δ* 174.3 (CO), 156.8 (C), 156.6 (C), 150.4 (C), 144.0 (CH), 140.0 (CH), 134.7 (CH), 133.9 (CH), 133.0 (CH), 131.1 (C), 128.9 (CH), 128.7 (CH), 128.4 (CH), 128.2 (C), 127.9 (CH), 116.5 (CH), 116.4 (CH), 114.5 (C), 114.3 (C), 110.9 (CH), 106.64 (CH), 106.63 (CH), 106.5 (CH), 25.78 (CH_3_), 25.01 (CH_3_) ppm, HRMS [M + H]^+^ calcd. for C_21_H_15_O_5_: 347.0914, measured: 347.0927.

#### 4/5-Methyl-3-oxo-3*H*-spiro[isobenzofuran-1,9′-xanthene]-3′,6′-diyl diacetate (8)

The mixture of 2-(6-hydroxy-3-oxo-3*H*-xanthen-9-yl)-4-methylbenzoic acid and 2-(6-hydroxy-3-oxo-3H-xanthen-9-yl)-5-methylbenzoic acid (3.0 g, 8.67 mmol) was treated with acetic anhydride (12.96 mL, 117.54 mmol, 13.5 equiv.) and heated at 140 °C for 3 h. After cooling down, 20 mL water and 40 mL toluene was added, and the mixture was evaporated to silica. The purification was performed by flash column chromatography using hexane-ethyl acetate as the eluents resulting in 2.38 g (5.53 mmol, 64%) of 4- and 5-methyl-3-oxo-3*H*-spiro[isobenzofuran-1,9′-xanthene]-3′,6′-diyl diacetate. ^1^H NMR (500 MHz, CDCl_3_) *δ* 7.89 (d, *J* = 7.9 Hz, 1H), 7.81 (s, 1H), 7.48 (d, *J* = 7.8 Hz, 1H), 7.41 (d, *J* = 7.9 Hz, 1H), 7.08 (d, *J* = 2.1 Hz, 4H), 7.06 (d, *J* = 7.9 Hz, 1H), 6.94 (s, 1H), 6.86 (s, 1H), 6.85–6.81 (m, 5H), 6.81–6.80 (m, 2H), 6.79 (d, *J* = 2.1 Hz, 1H), 2.51 (s, 3H), 2.39 (s, 3H), 2.30 (s, 6H), 2.29 (s, 6H) ppm, APT NMR (125 MHz, CDCl_3_) *δ* 172.5 (CO), 169.2 (CO), 168.8 (CO), 153.7 (C), 152.0 (C), 151.4 (C), 150.3 (C), 146.8 (C), 140.5 (C), 136.4 (CH), 131.2 (CH), 129.0 (CH), 128.9 (CH), 126.4 (C), 125.1 (CH), 124.9 (CH), 124.2 (CH), 123.7 (CH), 123.4 (C), 117.7 (CH), 117.6 (CH), 116.7 (C), 110.3 (CH), 22.0 (CH_3_), 21.3 (CH_3_), 21.1 (CH_3_) ppm, HRMS [M + H]^+^ calcd. for C_25_H_19_O_7_: 431.1125, measured: 431.1135.

#### 4/5-(Bromomethyl)-3-oxo-3*H*-spiro[isobenzofuran-1,9′-xanthene]-3′,6′-diyl diacetate (9)

The mixture of 4- and 5-methyl-3-oxo-3*H*-spiro[isobenzofuran-1,9′-xanthene]-3′,6′-diyl diacetate (8) (1.50 g, 3.49 mmol) was dissolved in 50 mL acetonitrile and heated to reflux. *N*-Bromosuccinimide (1.25 g, 7 mmol, 2 equiv.) and azobis-isobutyronitrile (171 mg, 1.05 mmol, 30%) was dissolved in 25 mL acetonitrile and added dropwise. After 24 h the dropwise addition was repeated, and the reaction mixture was stirred at room temperature for 48 h. The solvent was evaporated, the solid residue was dissolved in 100 mL dichloromethane and washed with water (2 × 30 mL). The organic phase was dried over MgSO_4_ and evaporated to silica. The crude product was purified by flash column chromatography using hexane-ethyl acetate as the eluent resulting in the mixture of 4- and 5-(bromomethyl)-3-oxo-3*H*-spiro[isobenzofuran-1,9′-xanthene]-3′,6′-diyl diacetate (9) as a red solid (0.88 g, 54%). ^1^H NMR (500 MHz, CDCl_3_) *δ* 8.02 (s, 0.5H), 7.98 (d, *J* = 8.0 Hz, 1H), 7.70 (dd, *J* = 8.0, 1.3 Hz, 0.5H), 7.66 (dd, *J* = 8.0, 0.9 Hz, 1H), 7.16 (s, 1H), 7.14 (s, 0.5H), 7.09 (s, 2H), 6.84–6.79 (m, 4H), 4.58 (s, 1H), 4.43 (s, 2H), 2.93 (s, 2H), 2.30 (s, 6H) ppm, APT NMR (125 MHz, CDCl_3_) *δ* 168.8 (CO), 168.3 (CO), 153.5 (C), 152.1 (C). 151.5 (C), 145.7 (C), 140.4 (C), 136.1 (CH), 131.2 (CH), 128.9 (CH), 126.8 (C), 125.9 (C), 125.7 (CH), 125.4 (CH), 124.5 (CH), 124.2 (CH), 117.9 (CH), 116.1 (C), 110.4 (CH), 31.3 (CH_2_), 28.7 (CH_2_), 21.1 (CH_3_) ppm, HRMS [M + H]^+^ calcd. for C_25_H_18_BrO_7_: 509.0230, measured 509.0258.

#### 4/5-(Aminomethyl)-2-(6-hydroxy-3-oxo-3*H*-xanthen-9-yl)benzoic acid (10)

The mixture of 4- and 5-(bromomethyl)-3-oxo-3*H*-spiro[isobenzofuran-1,9′-xanthene]-3′,6′-diyl diacetate (9) (0.85 g, 1.67 mmol) was dissolved in acetonitrile (10 mL) followed by the addition of 25 mL 25% aqueous ammonia in a sealed tube. The reaction mixture was kept at 35 °C for 1 h. The solvent was evaporated, and the crude product was triturated with water (2 × 5 mL) resulting in the mixture of 4- and 5-(aminomethyl)-2-(6-hydroxy-3-oxo-3H-xanthen-9-yl)benzoic acid (10) as red crystals (0.48 g, 79%). ^1^H NMR (500 MHz, DMSO-*d*_6_) *δ* 7.99 (s, 0.5 H), 7.90 (d, *J* = 7.9 Hz, 1H), 7.73 (d, *J* = 7.7 Hz, 0.5H), 7.64 (d, *J* = 8.0 Hz, 1H), 7.21 (s, 1H), 7.20 (d, *J* = 8.3 Hz, 0.5H), 6.65 (s, 3H), 6.57–6.49 (m, 6H), 3.96 (s, 1H), 3.85 (s, 2H) ppm, ^13^C NMR (125 MHz, DMSO-*d*_6_) *δ* 169.1 (CO), 161.0 (C), 160.7 (C), 153.0 (C), 152.6 (C), 152.4 (C), 135.5 (CH), 129.9 (CH), 129.6 (CH), 129.4 (CH), 125.8 (C), 125.1 (CH), 124.6 (CH), 124.4 (CH), 123.1 (CH), 113.6 (CH), 113.5 (CH), 113.3 (CH), 110.1 (C), 110.0 (C), 102.8 (CH), 102.7 (CH), 44.4 (CH_2_), 44.0 (CH_2_) ppm, HRMS [M + H]^+^ calcd. for C_21_H_16_NO_5_: 362.1022, measured: 362.1041.

#### 2-(6-Hydroxy-3-oxo-3*H*-xanthen-9-yl)-5-(isothiocyanatomethyl)-benzoic acid (4)

The mixture of 4- and 5-(aminomethyl)-2-(6-hydroxy-3-oxo-3*H*-xanthen-9-yl)benzoic acid (10) (100 mg, 0.28 mmol) was dissolved in DMSO (10 mL) and thiocarbonyldi-2(1*H*)-pyridone (75 mg, 0.30 mmol, 1.1 equiv.) was added at room temperature. The reaction was stirred for 20 min and then purified by preparative HPLC resulting in 2-(6-hydroxy-3-oxo-3*H*-xanthen-9-yl)-5-(isothiocyanatomethyl)benzoic acid (4) as a red solid (33 mg, 30%). ^1^H NMR (500 MHz, DMSO-*d*_6_) *δ* 8.05 (d, *J* = 8.0 Hz, 1H), 7.71 (d, *J* = 8.1 Hz, 1H), 7.25 (s, 1H), 6.73–6.66 (m, 4H), 6.62–6.54 (m, 6H), 5.07 (s, 2H) ppm, ^13^C NMR (125 MHz, DMSO-*d*_6_) *δ* 168.2 (CO), 159.5 (C), 153.3 (C), 151.8 (C), 142.7 (C), 137.1 (CH), 129.1 (CH), 125.9 (C), 125.4 (CH), 122.4 (CH), 112.7 (CH), 109.3 (C), 102.3 (CH), 82.9 (C), 47.7 (CH_2_) ppm, HRMS [M + H]^+^ calcd. for C_22_H_14_NO_5_S: 404.0587, measured: 404.0603.

## Conclusions

Through investigation of phenyl (1) and benzyl (2) isothiocyanates against surrogate thiol models we found that their reactivity and cysteine selectivity is different and pH-dependent. Considering the improved reactivity of 2 we hypothesized that fluorescent dyes equipped with the benzyl isothiocyanate functionality might have improved labelling efficiency. This has been confirmed in labelling studies using a set of proteins with catalytic and non-catalytic cysteines. We prepared the corresponding benzyl isothiocyanate derivative of fluorescein (4) and compared its spectrophotometric properties to that of the commonly used fluorescein isothiocyanate (3). These measurements revealed that 4 has improved quantum yield and brightness. This feature, together with the increased labelling efficiency of benzyl isothiocyanate, nominated 4 for antibody labelling studies on trastuzumab and its Fab. Our studies confirmed that FBITC (4) labels the cysteines of the Fab and the whole antibody more effectively than 3 and provides a stable conjugate independent from pH, and even under reducing conditions. These features suggest that FBITC (4) might be considered as a new fluorescent labelling agent with increased labelling efficiency and improved spectroscopic properties. As a further methodological and conceptual improvement, here a covalent fragment-based screening approach was used to identify a suitable cysteine-reactive warhead (BITC 2) leading to the development of an improved fluorophore (FBITC 4). Actually the engagement of the covalent fragment theory with the antibody conjugation techniques provided here valuable development in the field of fluorescent dye chemistry. We believe a similar high-throughput (for an academic environment) approach could be used to develop further protein-modification tools.

## Conflicts of interest

There are no conflicts to declare.

## Supplementary Material

RA-010-D0RA02934C-s001
