# Supplementary material for: Cysteine specific bioconjugation with benzyl isothiocyanates
Source: RSC Adv. 2020 Apr 16;10(25):14928–36. doi: 10.1039/d0ra02934c (PMC9052032; doi:10.1039/d0ra02934c)

Electronic Supplementary Material (ESI) for RSC Advances.
This journal is © The Royal Society of Chemistry 2020

**Electronic Supplementary Information**

# Cysteine specific bioconjugation with benzyl isothiocyanates

László Petri,[a] Péter A. Szijj,[b] Ádám Kelemen,[a] Tímea Imre,[c] Ágnes Gömöry,[d] Maximillian T. W. Lee,[b] Krisztina Hegedűs,[e] Péter Ábrányi-Balogh,[a] Vijay Chudasama *[b] and György Miklós Keserű *[a]

[a] Research Centre for Natural Sciences, Medicinal Chemistry Research Group, H-1117, Budapest, Magyar tudósok krt 2, HU.
[b] Department of Chemistry, University College London, 20 Gordon Street, London WC1H OAJ, UK.
[c] Research Centre for Natural Sciences, MS Metabolomics Research Group, H-1117, Budapest, Magyar tudósok krt 2, HU.
[d] Research Centre for Natural Sciences, MS Proteomics Research Group, H-1117, Budapest, Magyar tudósok krt 2, HU.
[e] Eötvös Loránd University Department of Immunology, H-1117 Budapest, Pázmány Péter sétány 1/C.

## Table of Contents

**Figure S1.** MS analysis of tau protein labelling with fragments **1** and **2**. ....2

**Figure S2.** $^{15}$N-HSQC NMR analysis of KRas G12C protein labelling with fragments **1** and **2**. ....3

**Figure S3.** Identification and analysis of trastuzumab Fab ....4

**Figure S4.** Labelling of reduced Fab at pH = 6.5 with **1** fragment (1000 eq.) ....5

**Figure S5.** Labelling of reduced Fab at pH = 8.0 with **1** fragment (1000 eq.) ....5

**Figure S6.** Labelling of reduced Fab at pH = 6.5 with **2** fragment (1000 eq.) ....6

**Figure S7.** Labelling of reduced Fab at pH = 8.0 with **2** fragment (1000 eq.) ....6

**Figure S8.** Labelling of native Fab at pH = 6.5 with **2** fragment (1000 eq.) ....7

**Figure S9.** Labelling of native Fab at pH = 8.0 with **2** fragment (1000 eq.) ....7

**Figure S10.** Labelling of reduced Fab at pH = 6.5 with **3** dye (10 eq.) ....8

**Figure S11.** Labelling of native Fab at pH = 6.5 with **3** dye (10 eq.) ....9

**Figure S12.** Labelling of reduced Fab at pH = 8.0 with **3** dye (10 eq.) ....9

**Figure S13.** Labelling of native Fab at pH = 8.0 with **3** dye (10 eq.) ....10

**Figure S14.** Labelling of reduced Fab at pH = 6.5 with **4** dye (10 eq.) ....11

**Figure S15.** Labelling of native Fab at pH = 6.5 with **4** dye (10 eq.) ....12

**Figure S16.** Labelling of reduced Fab at pH = 8.0 with **4** dye (10 eq.) ....13

**Figure S17.** Labelling of native Fab at pH = 8.0 with **4** dye (10 eq.) ....13

**Figure S18.** Stability of the **4** dye labelled reduced Fab at pH = 6.8 ....14

**Figure S19.** Stability of the **4** dye labelled reduced Fab at pH = 7.4 ....15

**Figure S20.** Stability of the **4** dye labelled reduced Fab at pH = 8.0 ....16

**Figure S21.** Stability of the **4** dye labelled reduced Fab at pH = 7.4, with 5 µM GSH ....17

**Figure S22.** Analysis of reduced and partially re-oxidized trastuzumab (re-oxidation over 5 h, 4 °C) ..18

**Figure S23.** Identification and analysis of trastuzumab bioconjugation with **3** dye, at pH = 8.0 ....19

**Figure S24.** Identification and analysis of trastuzumab bioconjugation with **4** dye, at pH = 8.0 ....20

**NMR spectra of synthesized compounds** ....21

**Figure S1.** MS analysis of tau protein labelling with fragments **1** and **2**.

**a)** Deconvoluted mass spectra of the tau protein

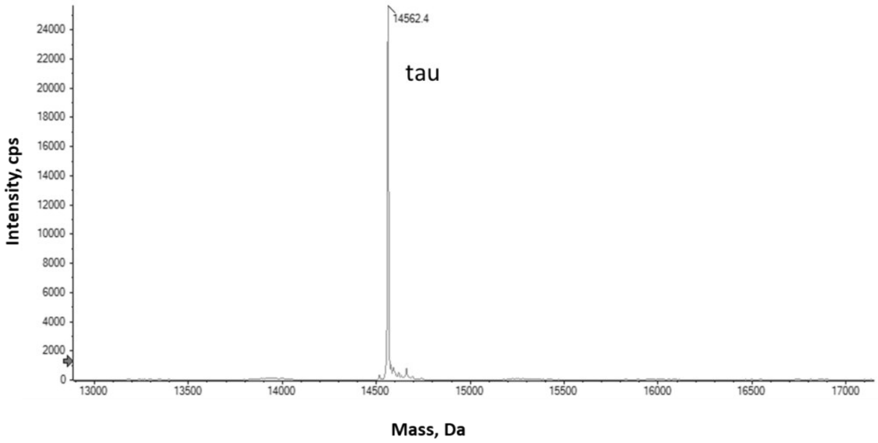


**b)** Deconvoluted mass spectra of the tau protein labelled with with phenyl-isothiocyanate (**1**).

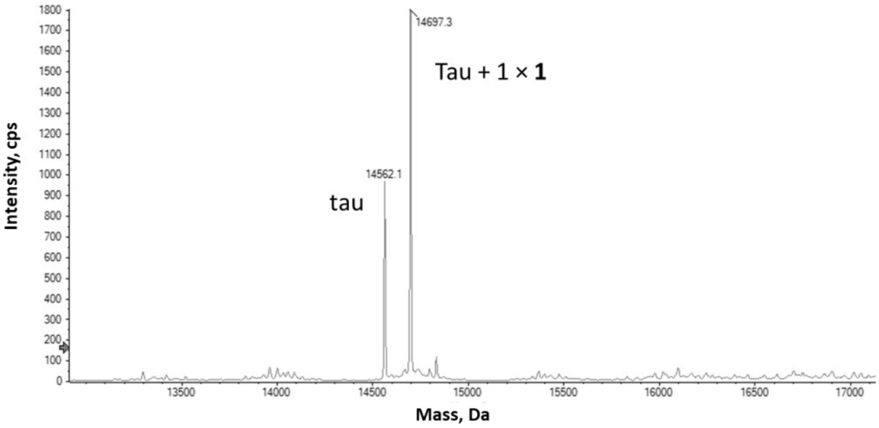


Single labelling was detected, corresponding to the observed mass shift ($M_w$ = 14 697 Da). Noteworthy, unlabelled tau protein was also observed ($M_w$ = 14 562 Da).

**c)** Deconvoluted mass spectra of the tau protein labelled with with benzyl-isothiocyanate (**2**).

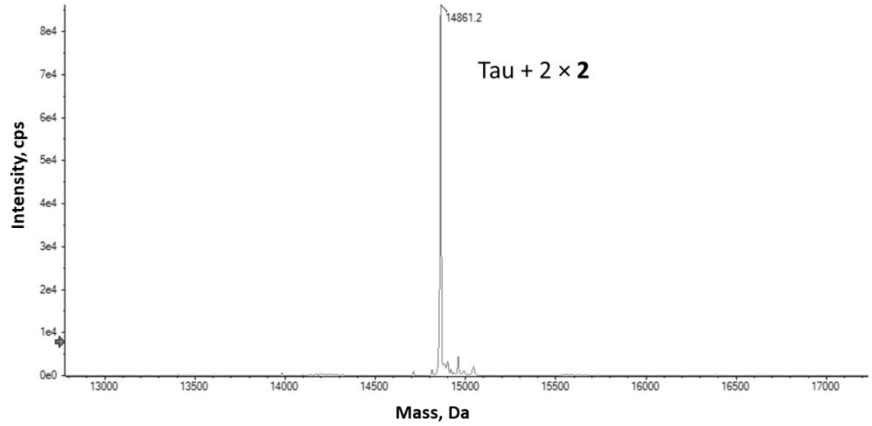


Double labelling was detected, corresponding to the observed mass shift ($M_w$ = 14 861 Da).

**Figure S2.** $^{15}$N-HSQC NMR analysis of KRas G12C protein labelling with fragments **1** and **2**.

**a)** $^{15}$N-HSQC NMR analysis of KRas G12C protein labelled with phenyl-isothiocyanate (**1**).

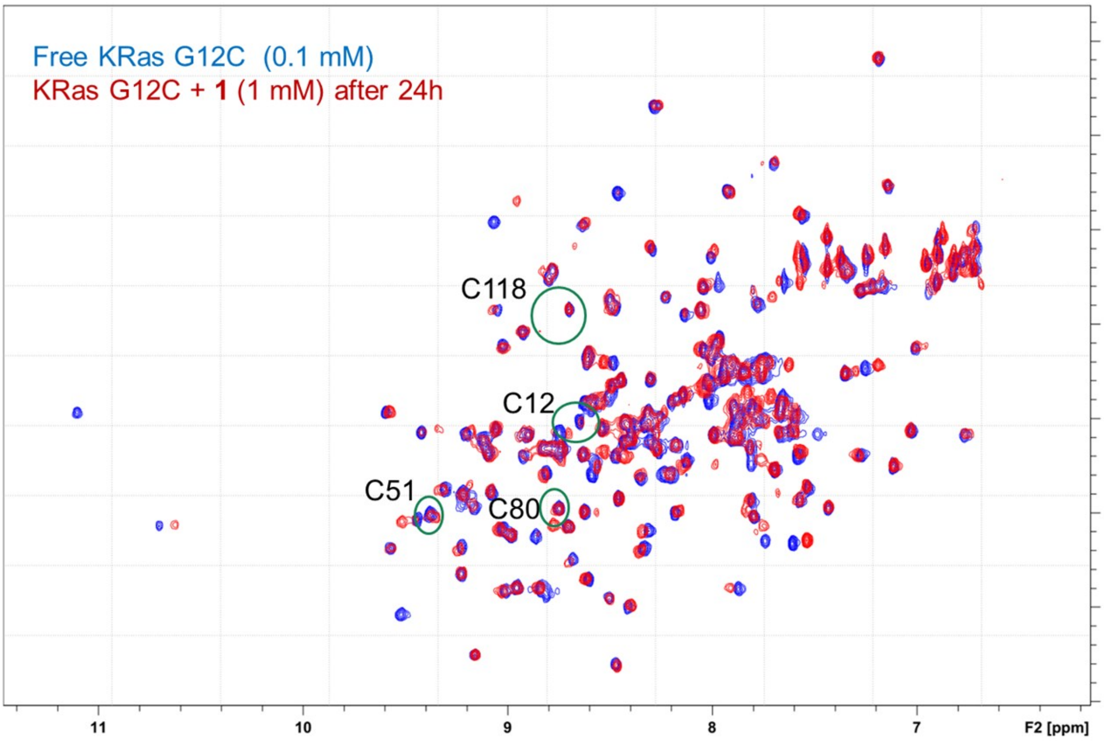


Peak changes were detected correspoding only to the *Cys12*, however, based on the relative integral values, the labelling reached 54% conversion after 24h incubation.

**b)** $^{15}$N-HSQC NMR analysis of KRas G12C protein labelled with benzyl-isothiocyanate (**2**).

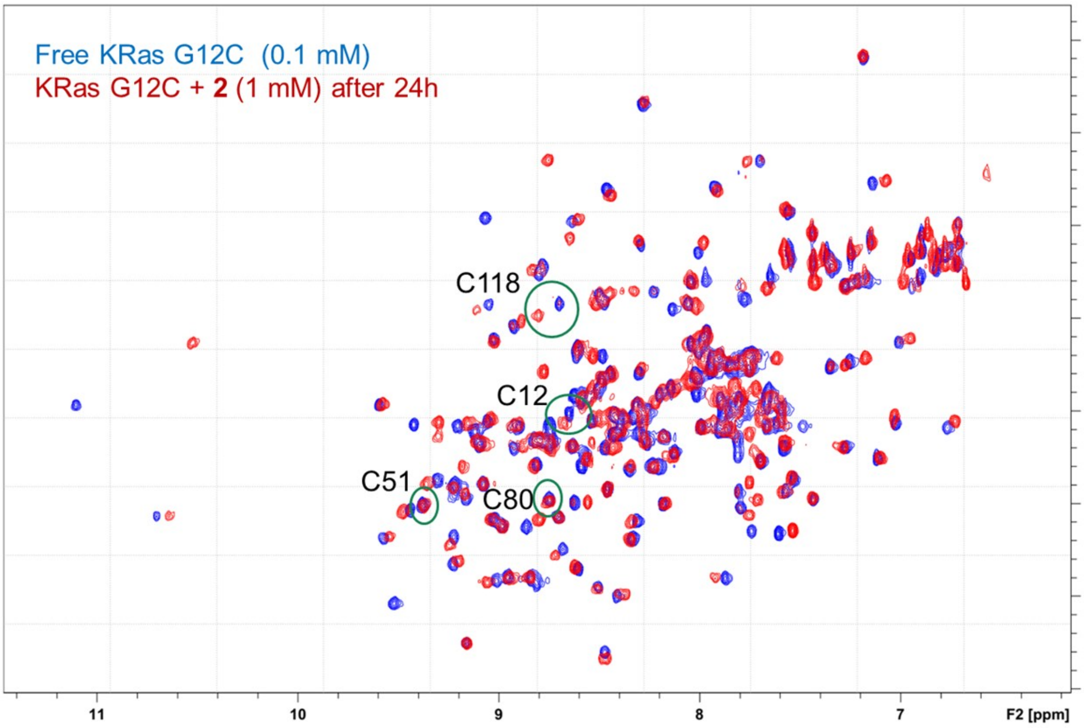


Peak changes were detected correspoding to the *Cys12* and *Cys118*, furthermore, based on the relative integral values, the labelling of the residues reached 98% conversion after 24h incubation.

**Figure S3.** Identification and analysis of trastuzumab Fab

Expected mass: 47638 Da. Observed mass: 47639 Da.

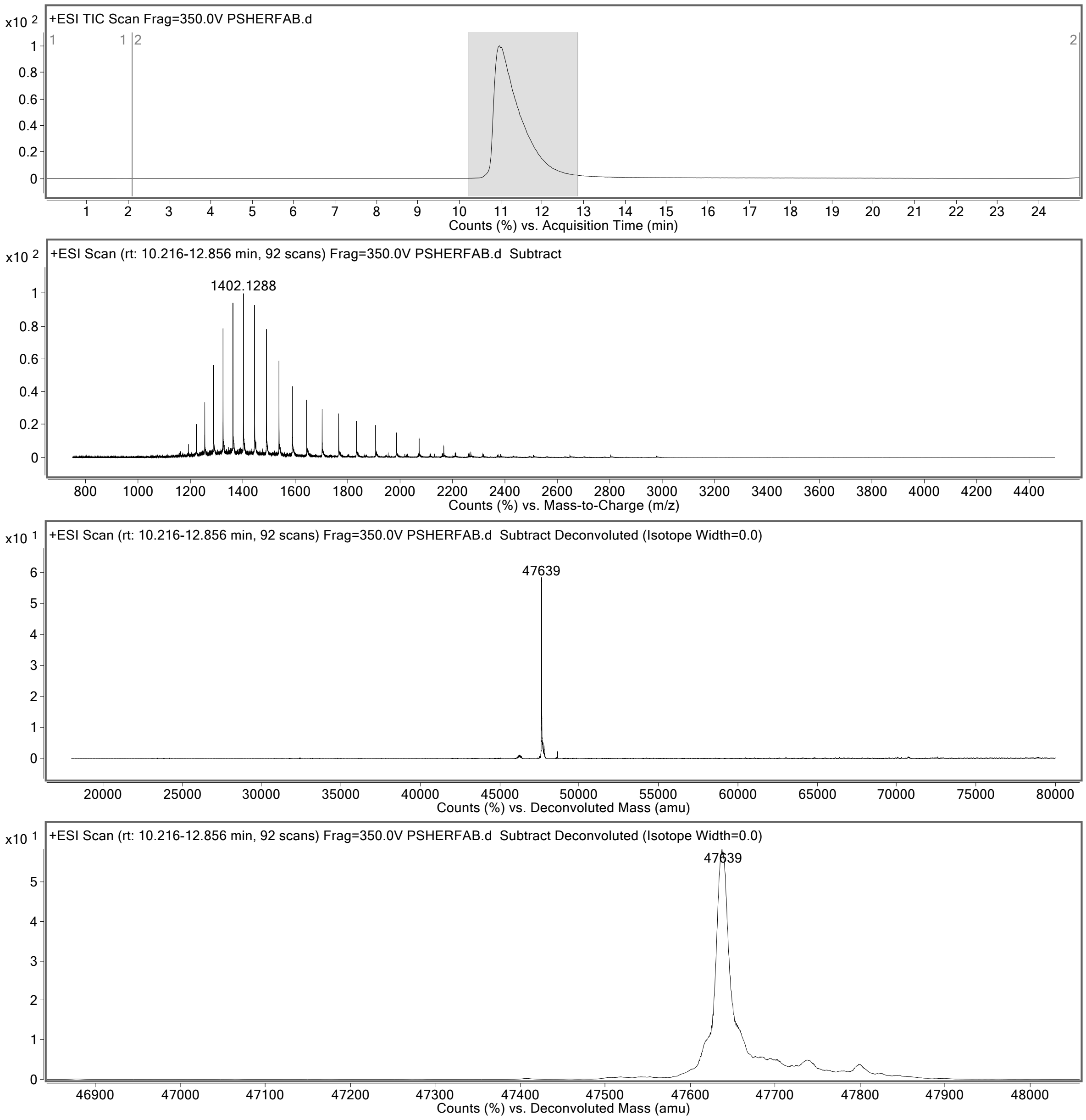

**Figure S4.** Labelling of reduced Fab at pH = 6.5 with **1** fragment (1000 eq.)

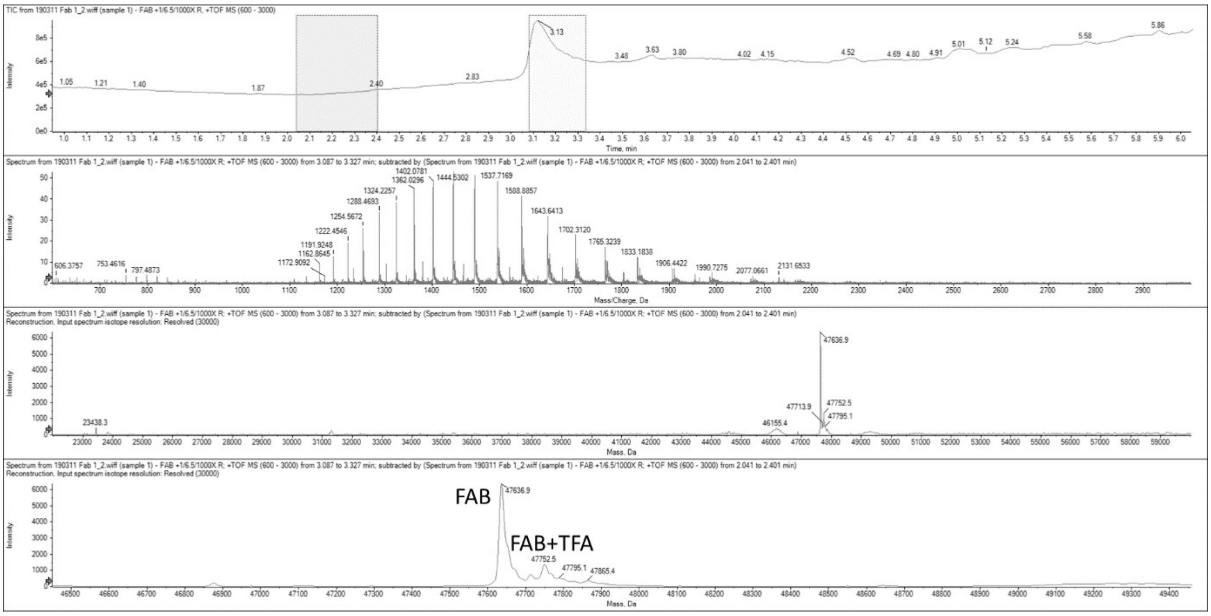


**Figure S5.** Labelling of reduced Fab at pH = 8.0 with **1** fragment (1000 eq.)

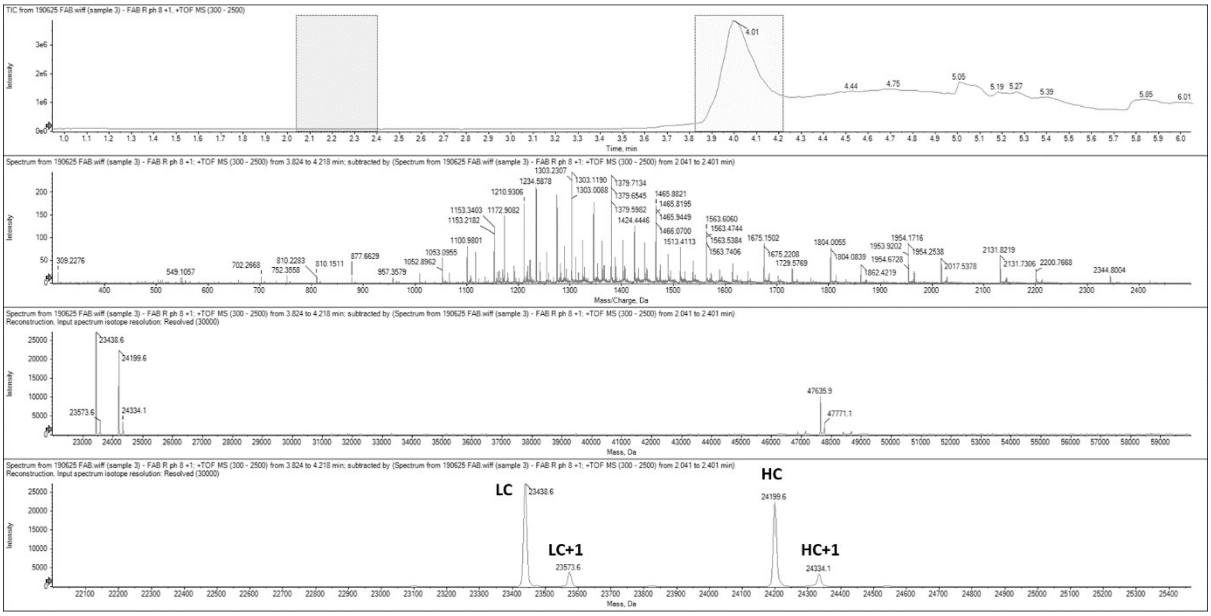

**Figure S6.** Labelling of reduced Fab at pH = 6.5 with **2** fragment (1000 eq.)

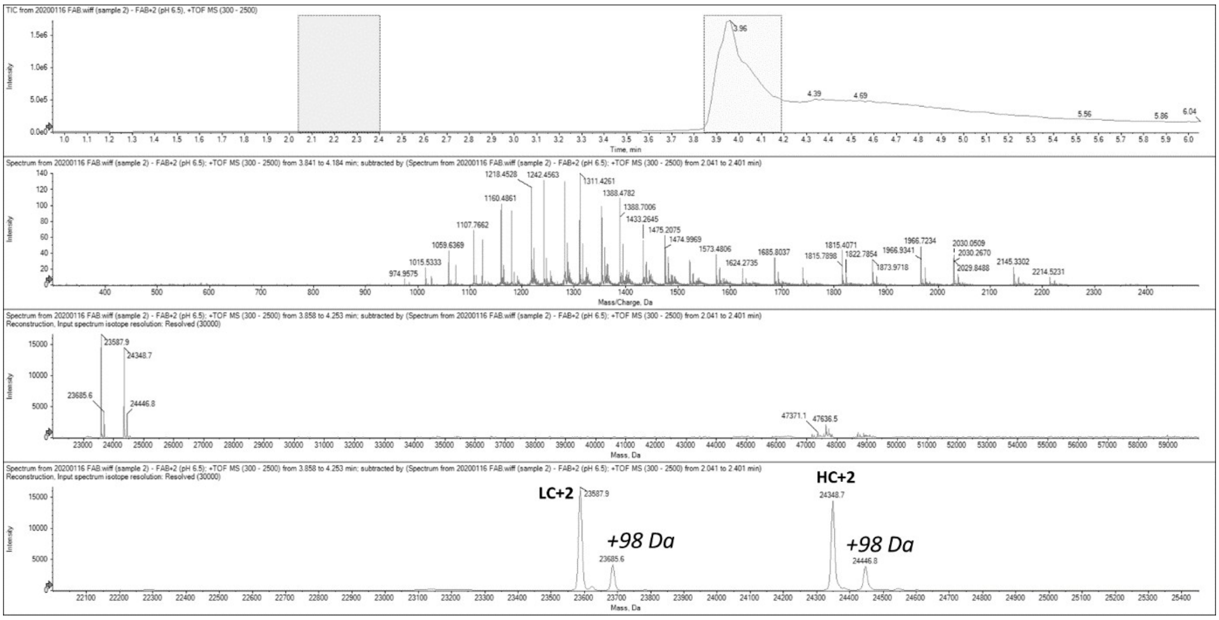


**Figure S7.** Labelling of reduced Fab at pH = 8.0 with **2** fragment (1000 eq.)

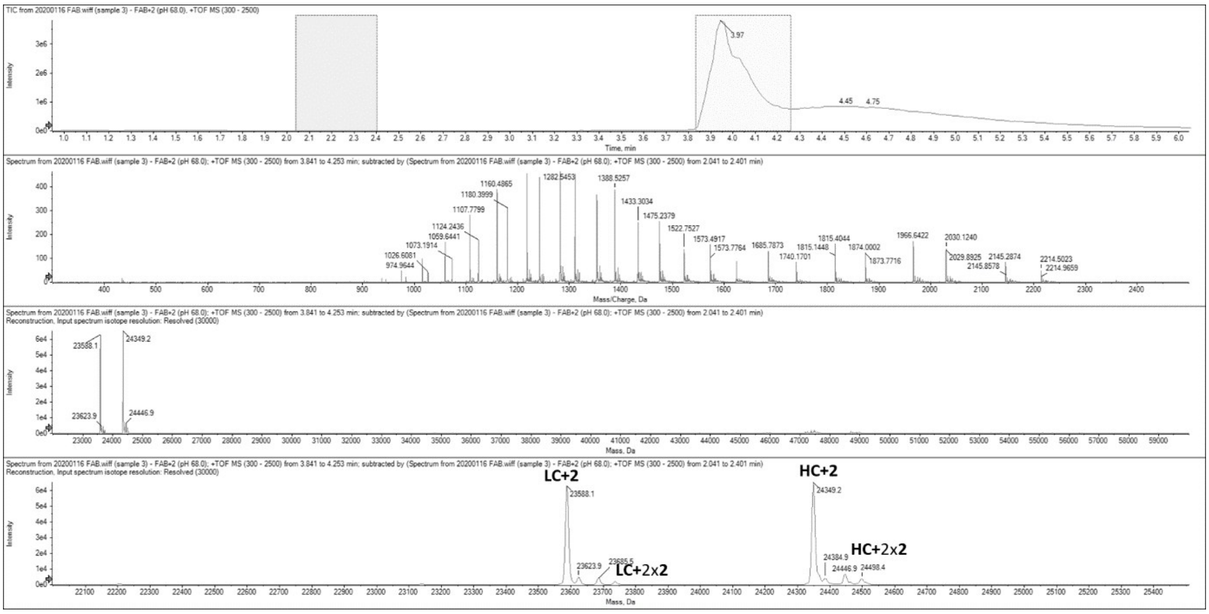

**Figure S8.** Labelling of native Fab at pH = 6.5 with **2** fragment (1000 eq.)

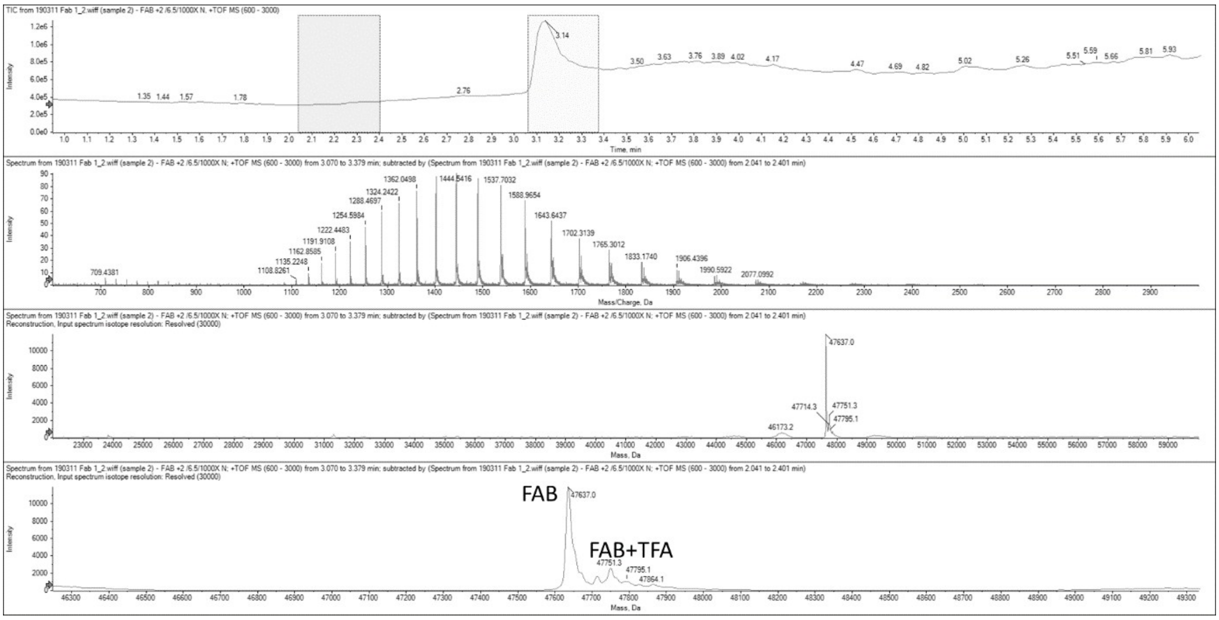


**Figure S9.** Labelling of native Fab at pH = 8.0 with **2** fragment (1000 eq.)

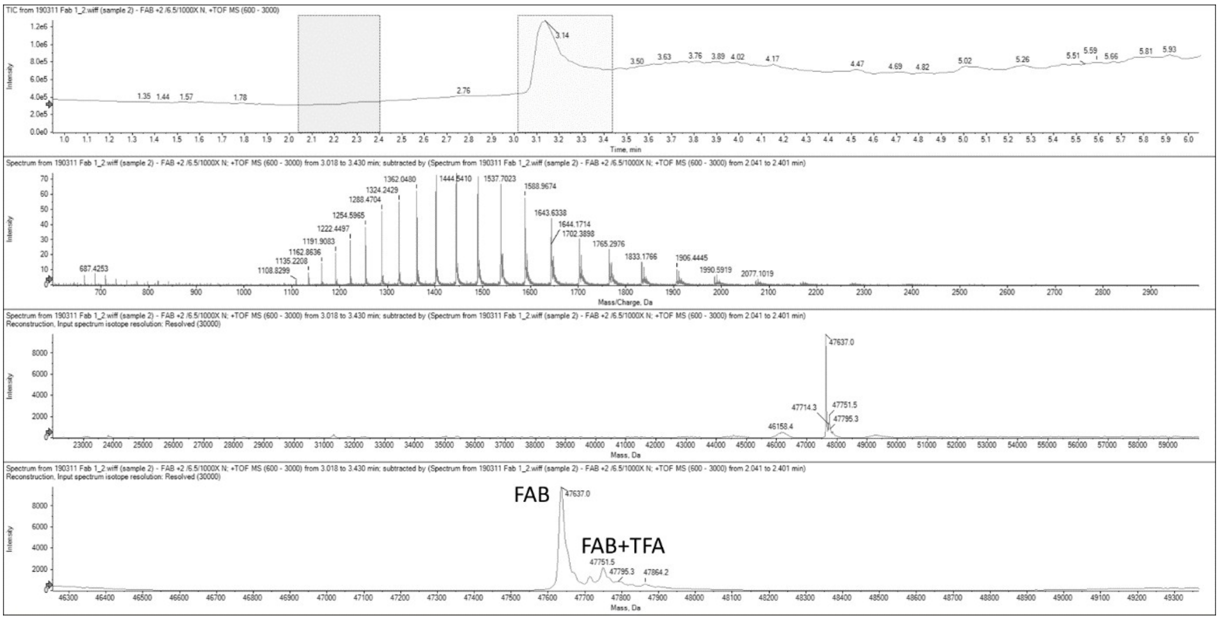

**Figure S10.** Labelling of reduced Fab at pH = 6.5 with **3** dye (10 eq.)

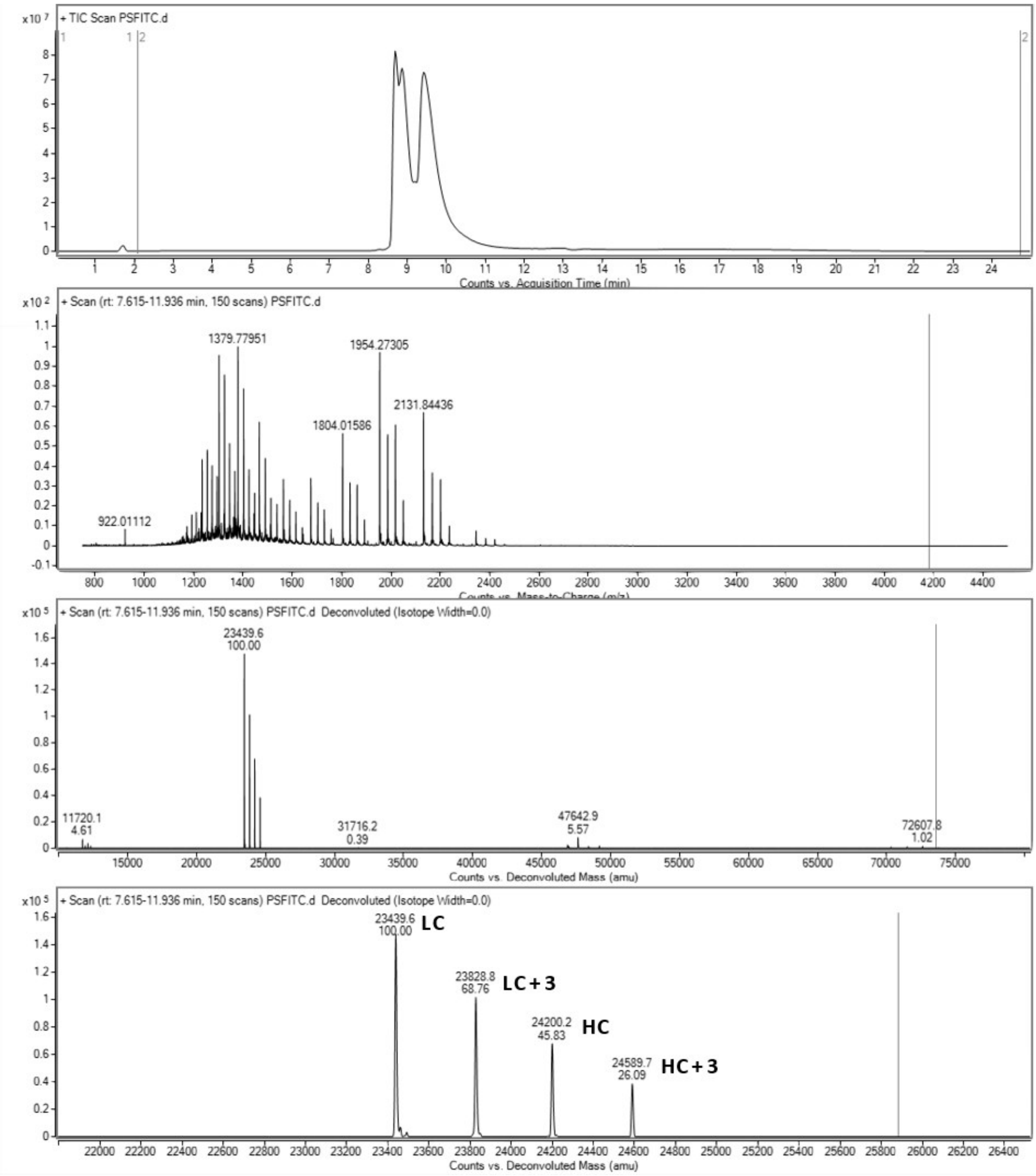

**Figure S11.** Labelling of native Fab at pH = 6.5 with **3** dye (10 eq.)

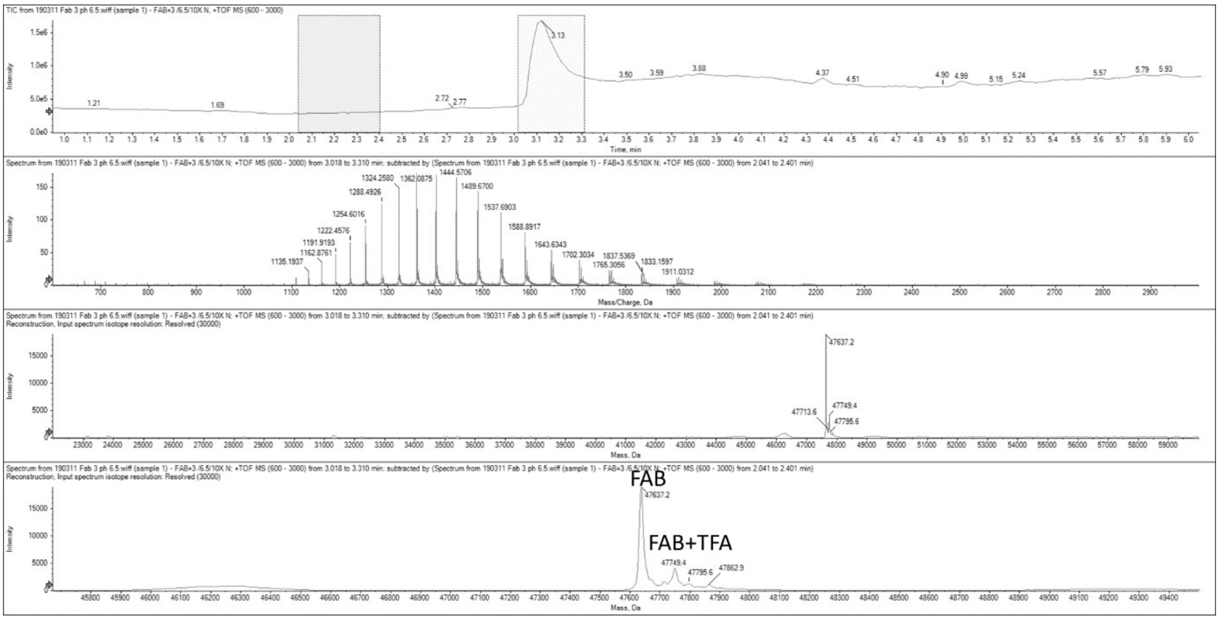


**Figure S12.** Labelling of reduced Fab at pH = 8.0 with **3** dye (10 eq.)

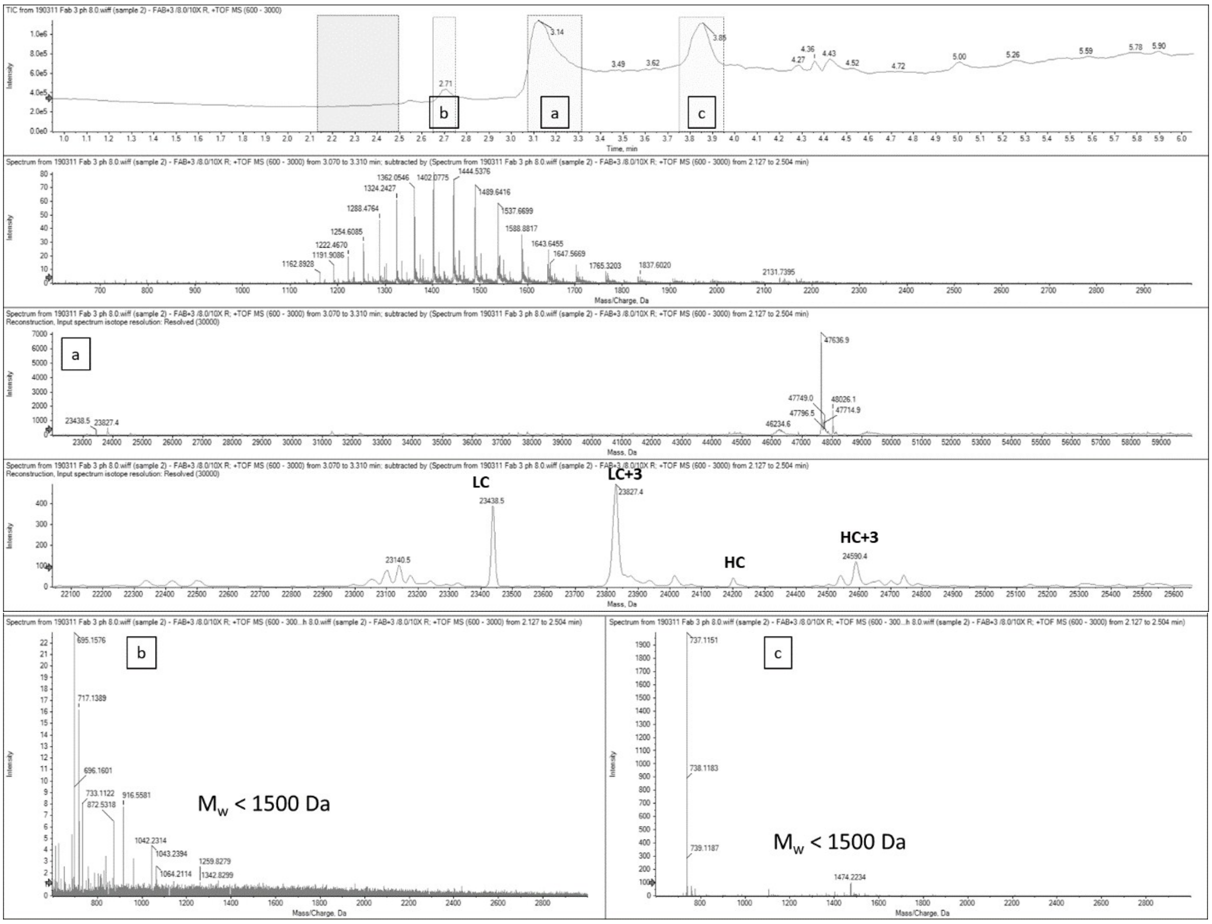

**Figure S13.** Labelling of native Fab at pH = 8.0 with **3** dye (10 eq.)

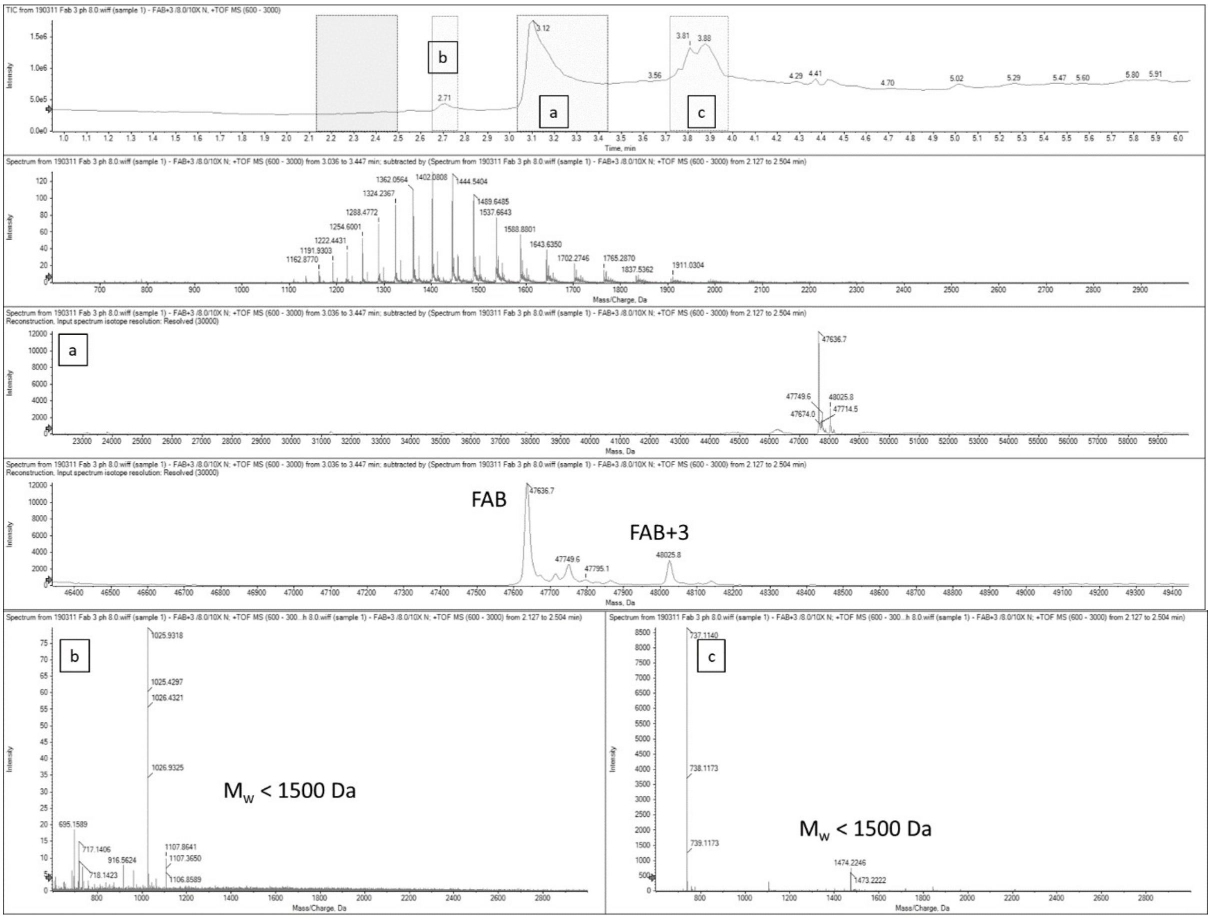

**Figure S14.** Labelling of reduced Fab at pH = 6.5 with **4** dye (10 eq.)

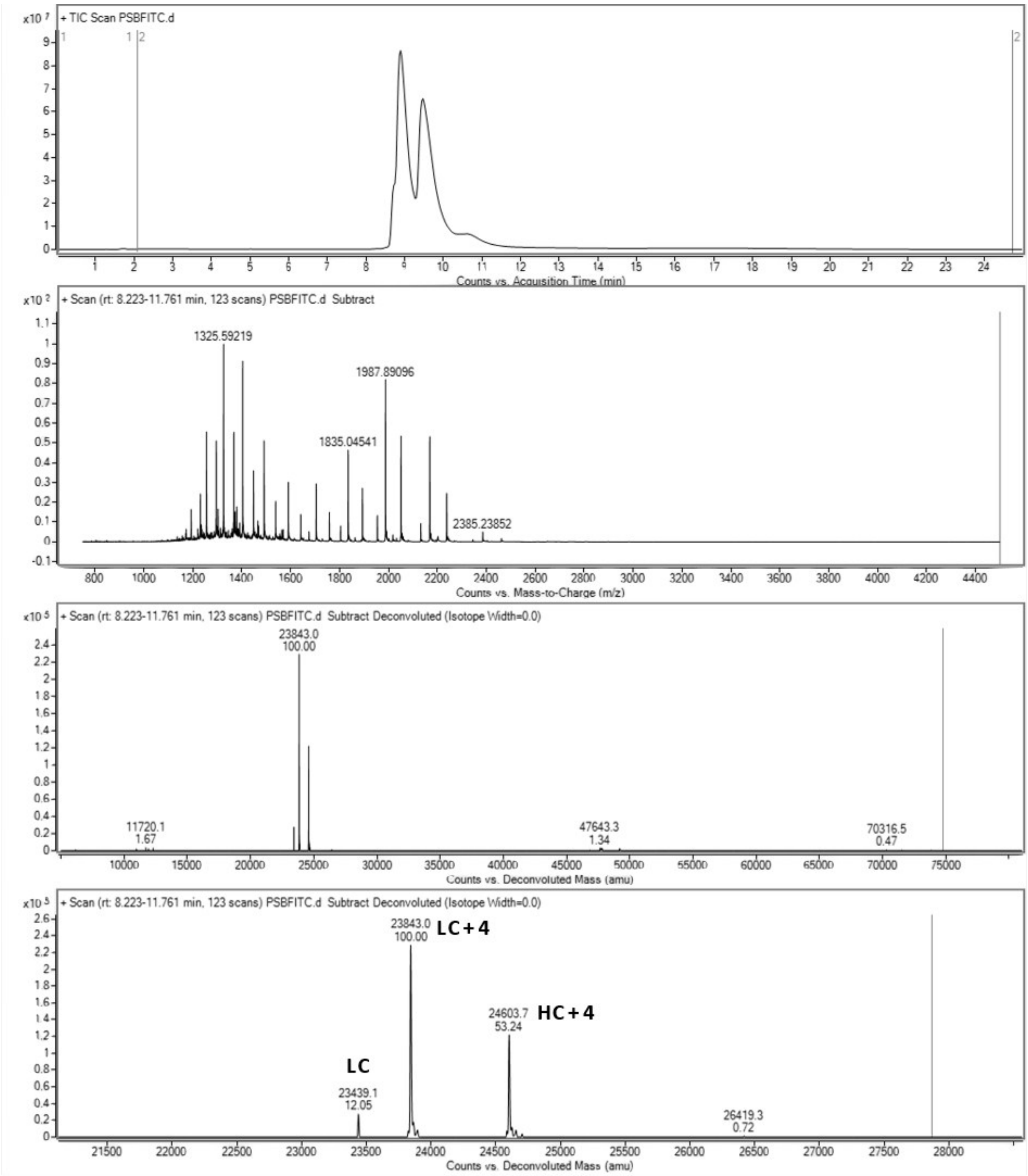

**Figure S15.** Labelling of native Fab at pH = 6.5 with **4** dye (10 eq.)

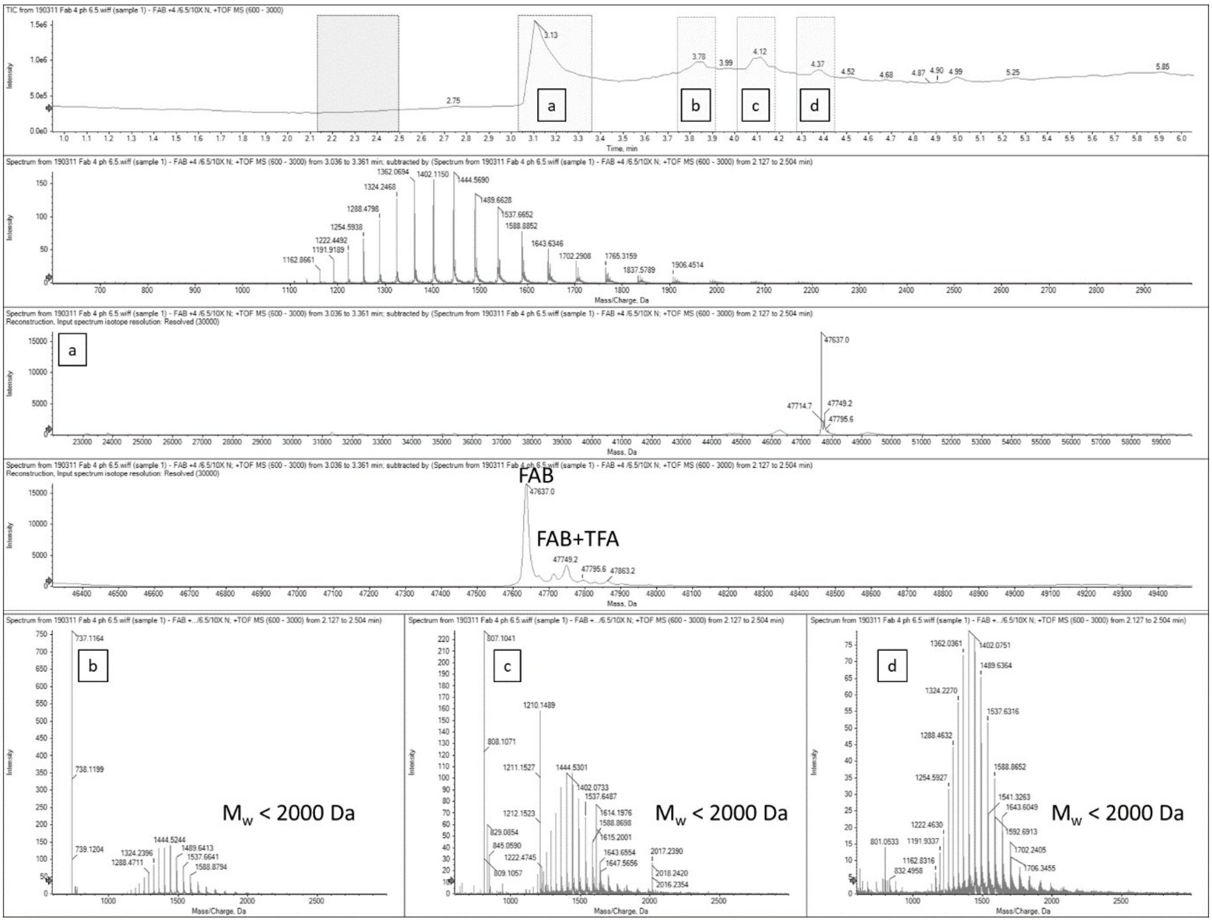

**Figure S16.** Labelling of reduced Fab at pH = 8.0 with **4** dye (10 eq.)

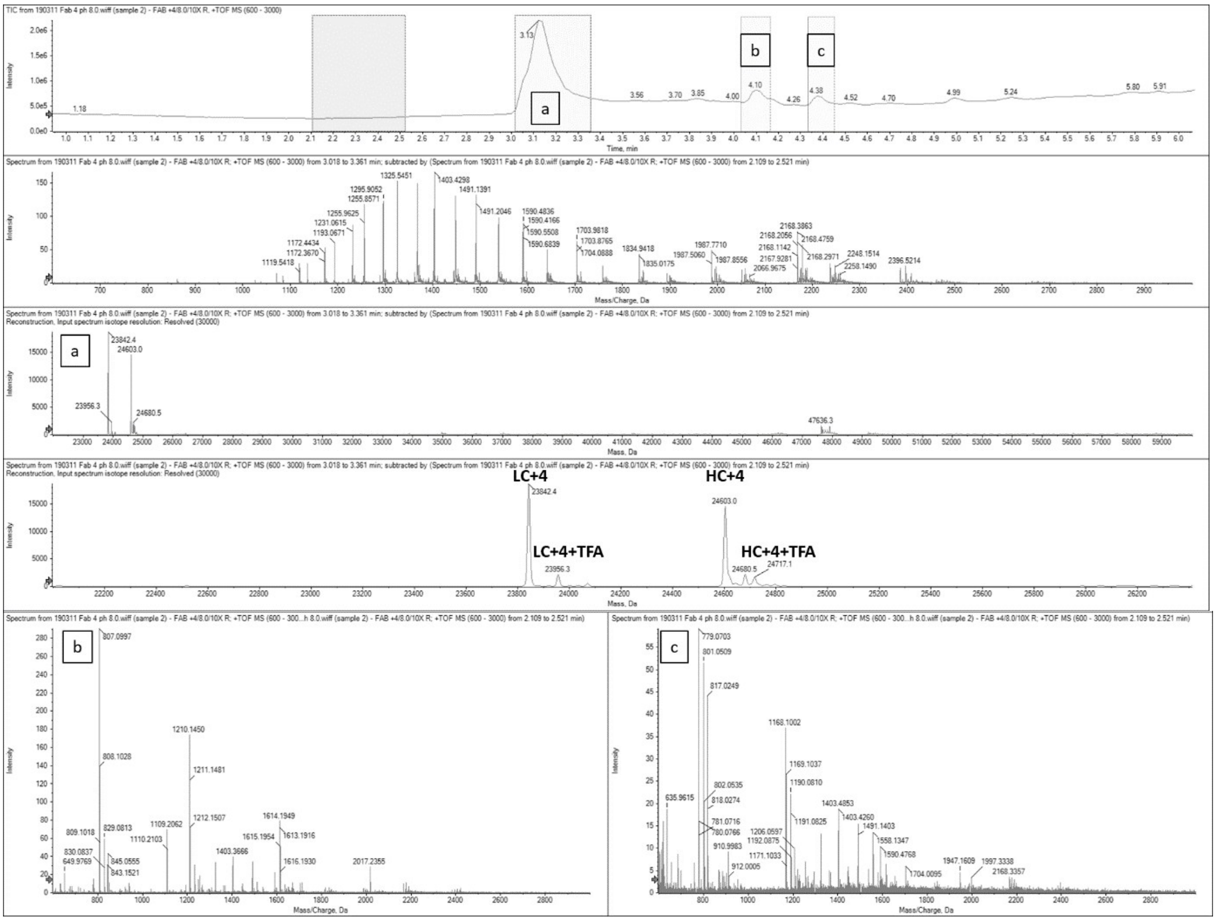


**Figure S17.** Labelling of native Fab at pH = 8.0 with **4** dye (10 eq.)

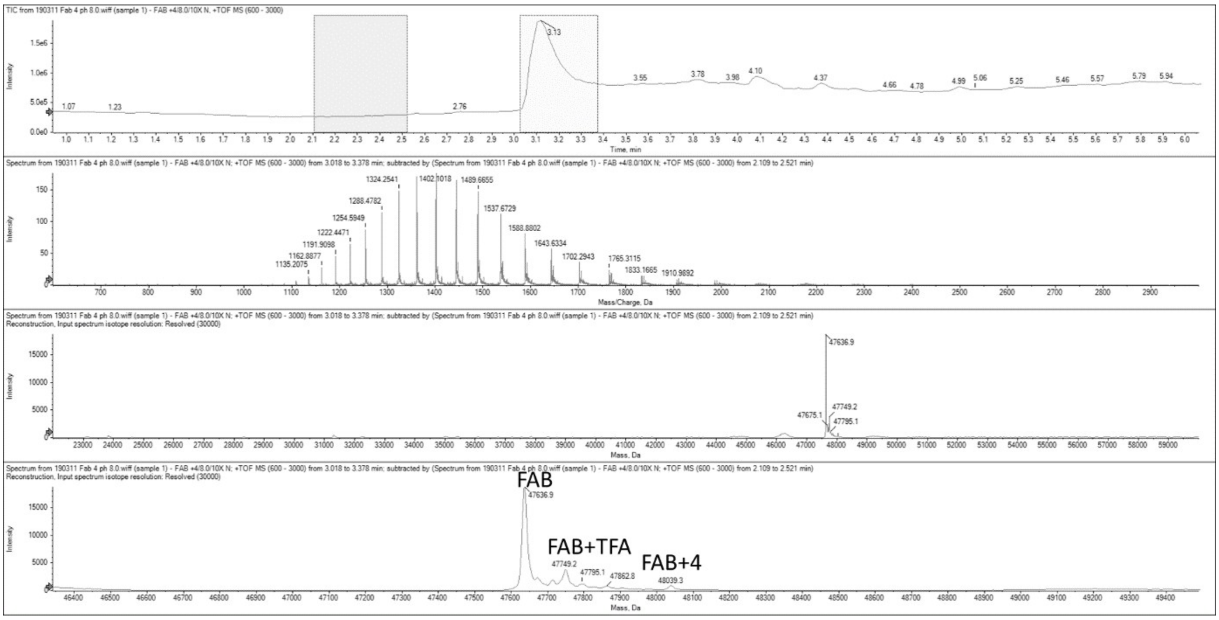

**Figure S18.** Stability of the **4** dye labelled reduced Fab at pH = 6.8

**a)** MS analysis after 0h

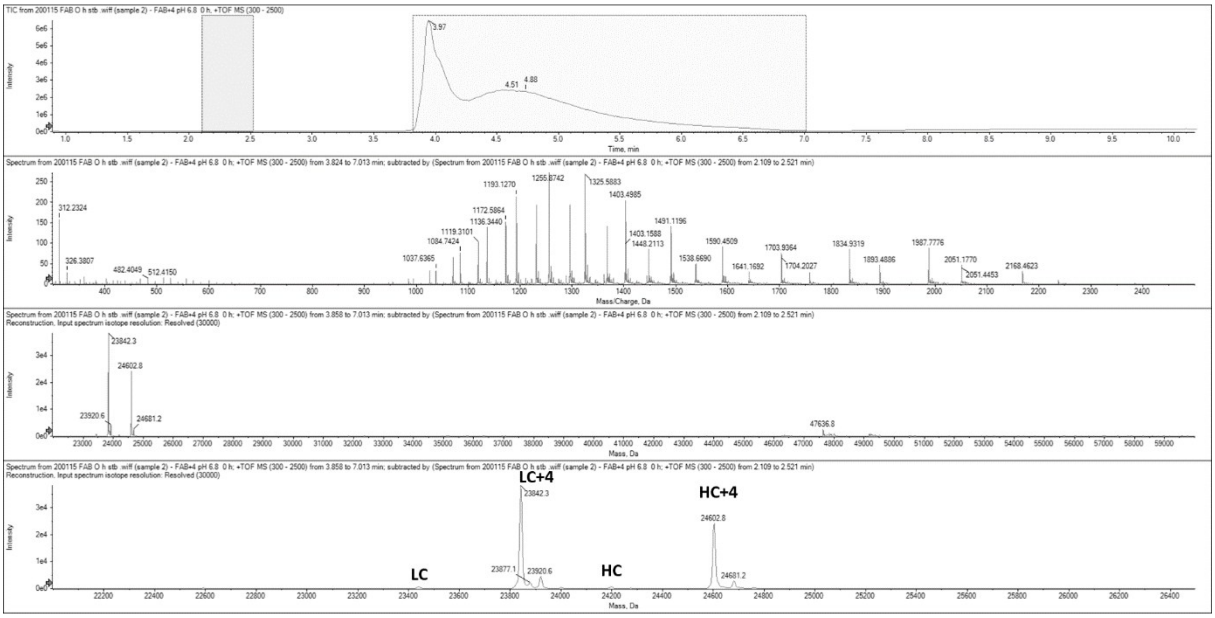


**b)** MS analysis after 24h

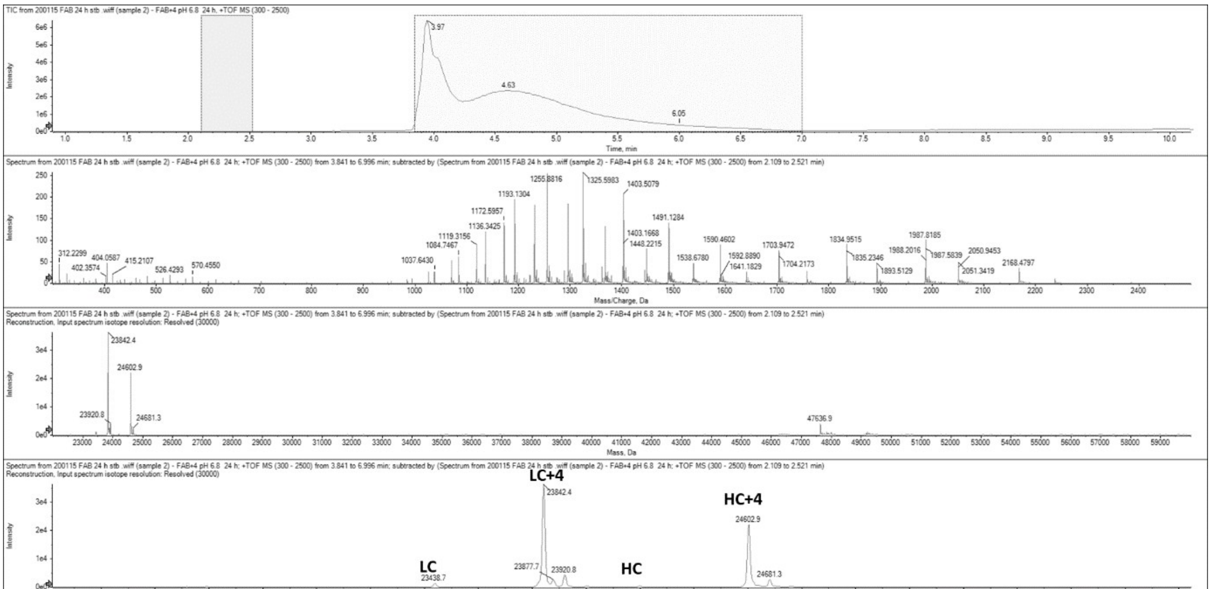

**Figure S19.** Stability of the **4** dye labelled reduced Fab at pH = 7.4

**a)** MS analysis after 0h

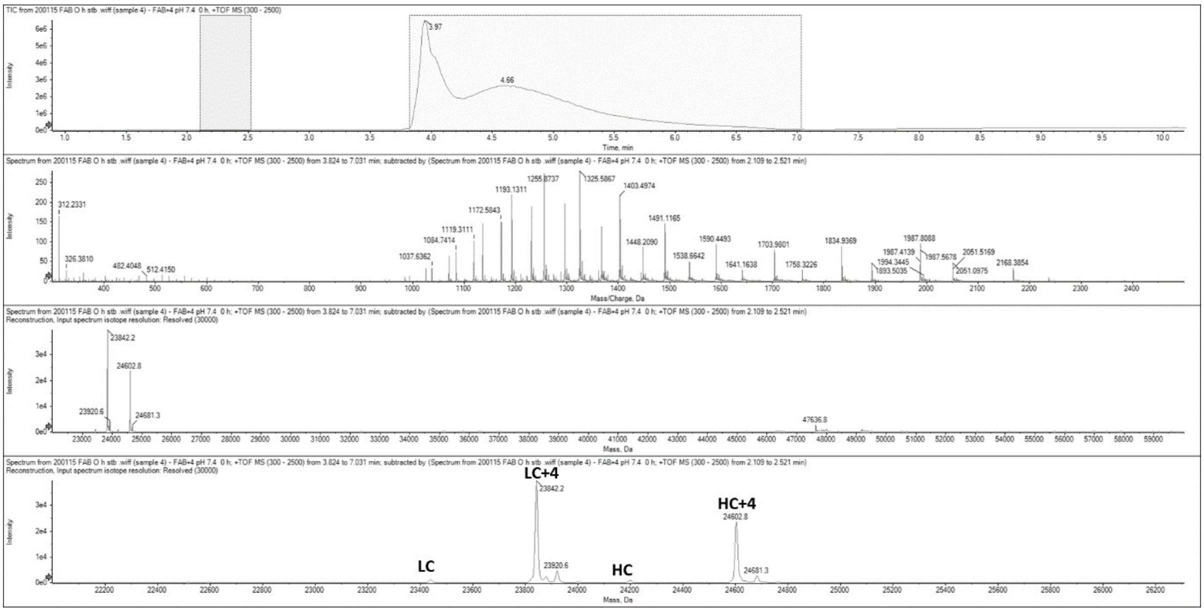


**b)** MS analysis after 24h

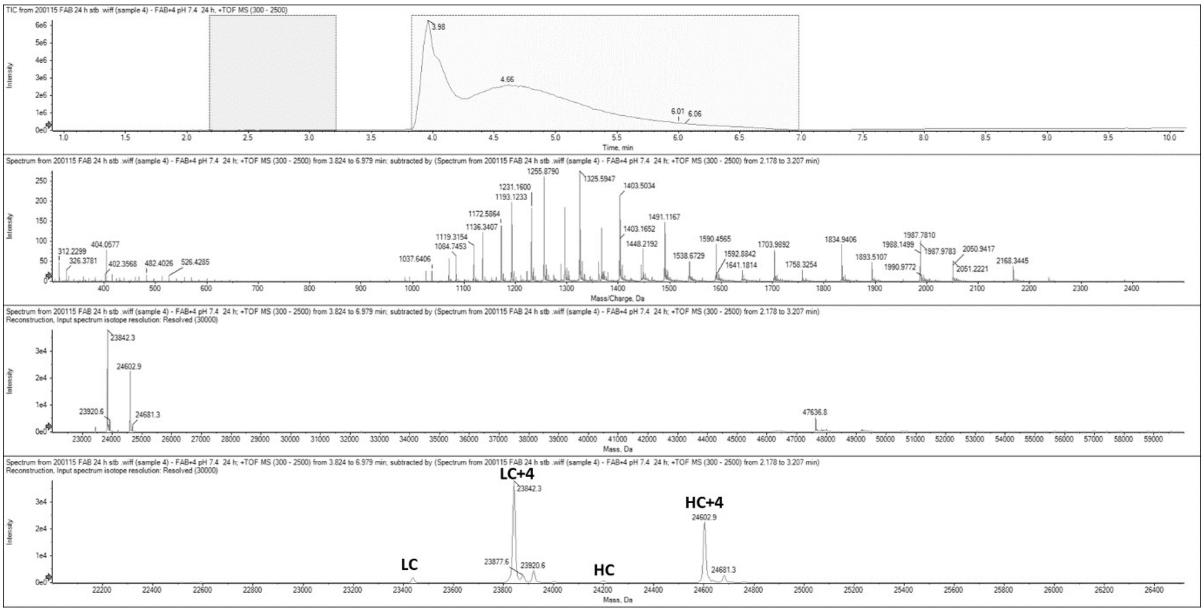

**Figure S20.** Stability of the **4** dye labelled reduced Fab at pH = 8.0

**a)** MS analysis after 0h

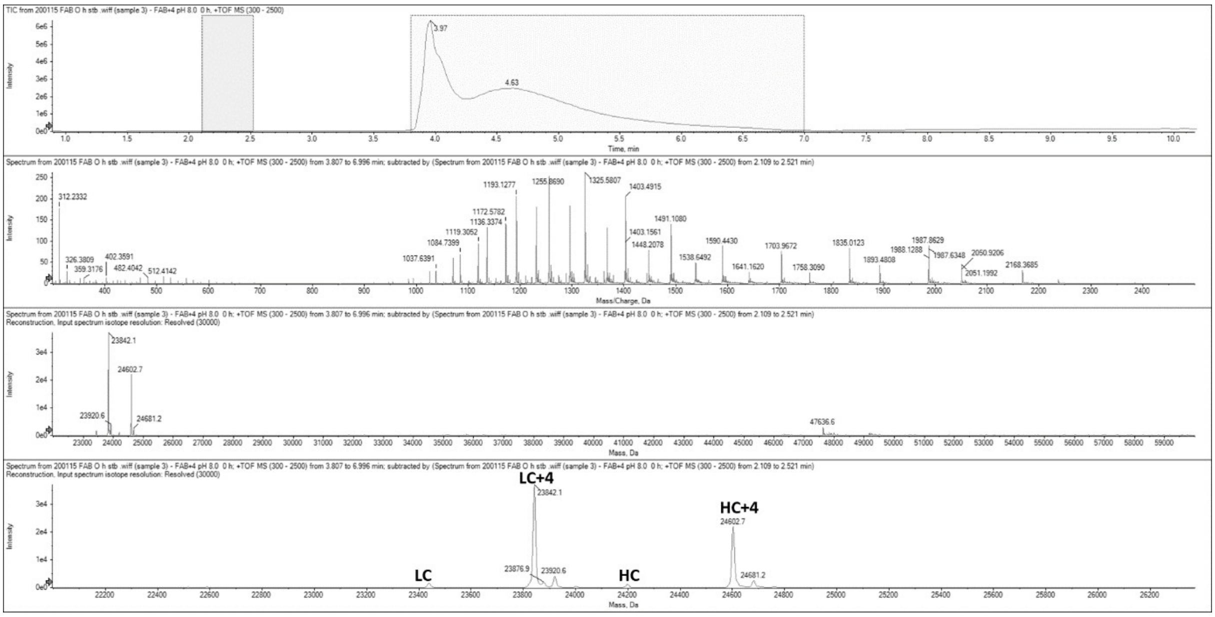


**b)** MS analysis after 24h

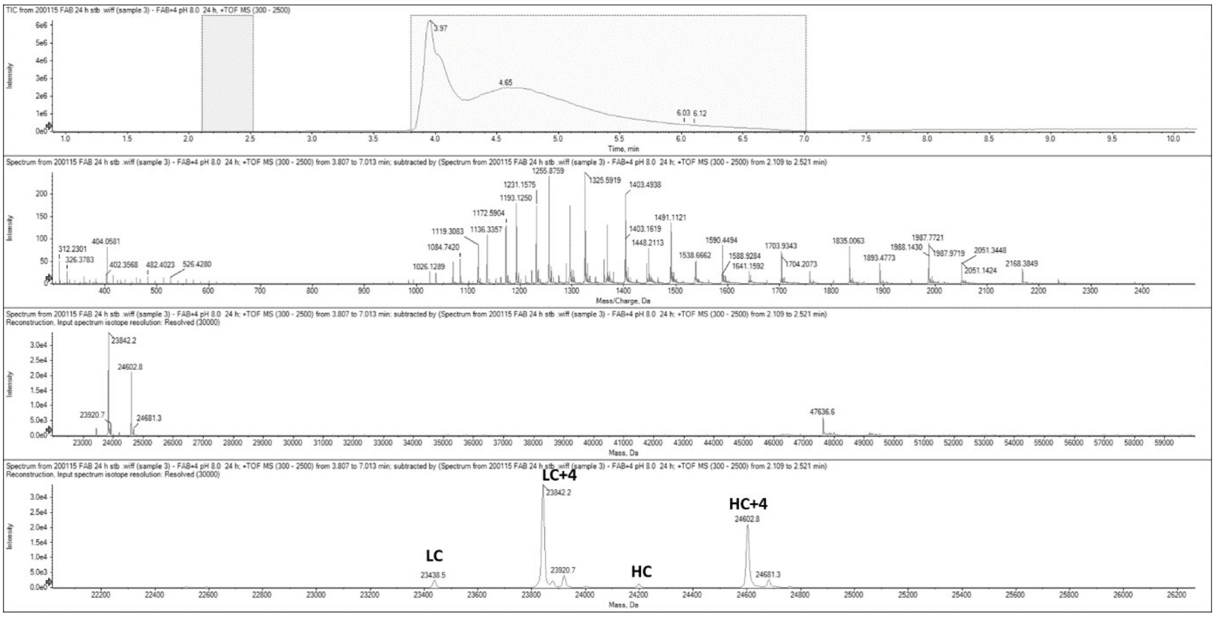

**Figure S21.** Stability of the **4** dye labelled reduced Fab at pH = 7.4, with 5 µM GSH

**a)** MS analysis after 0h

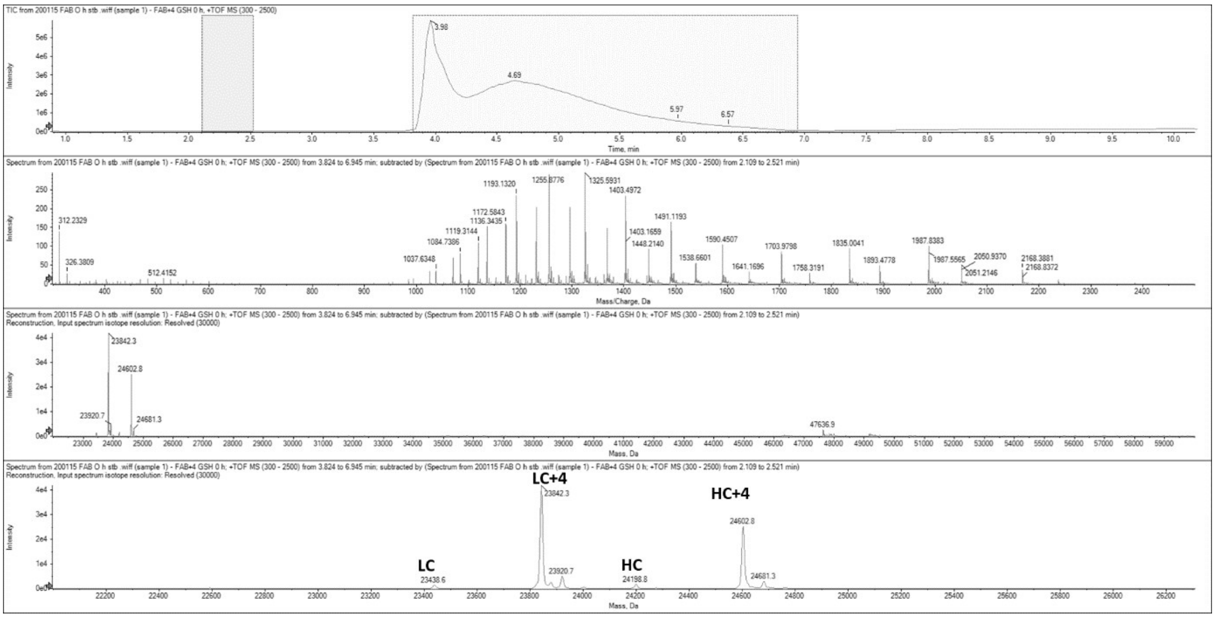


**b)** MS analysis after 24h

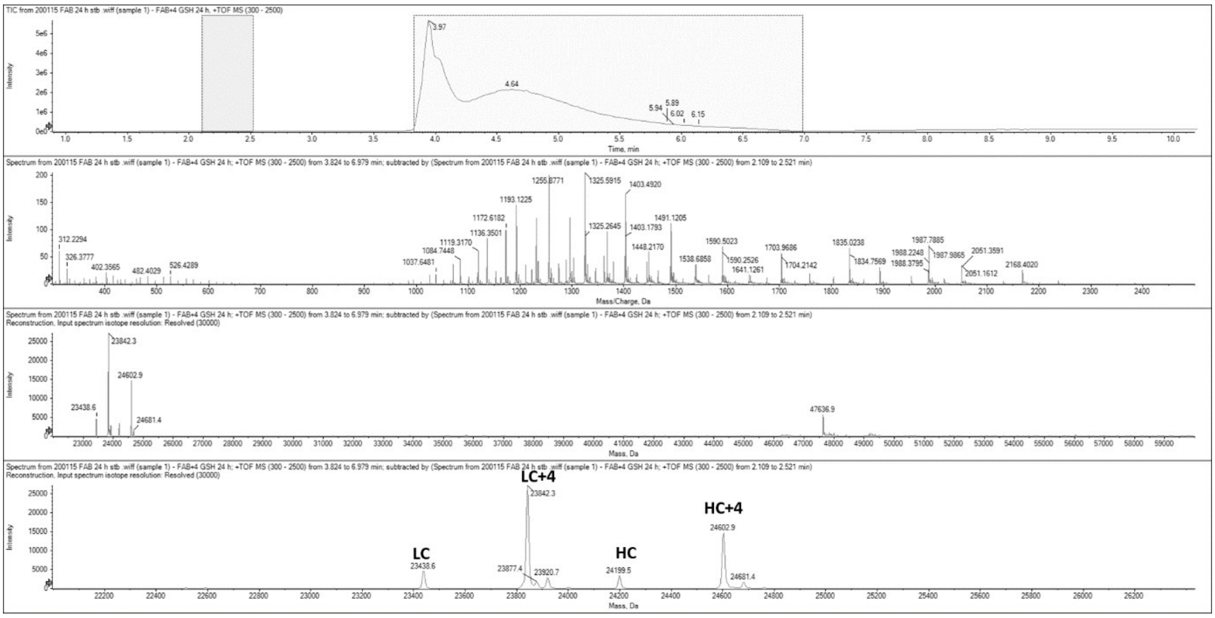

**Figure S22.** Analysis of reduced and partially re-oxidized trastuzumab (re-oxidation over 5 h, 4 °C)

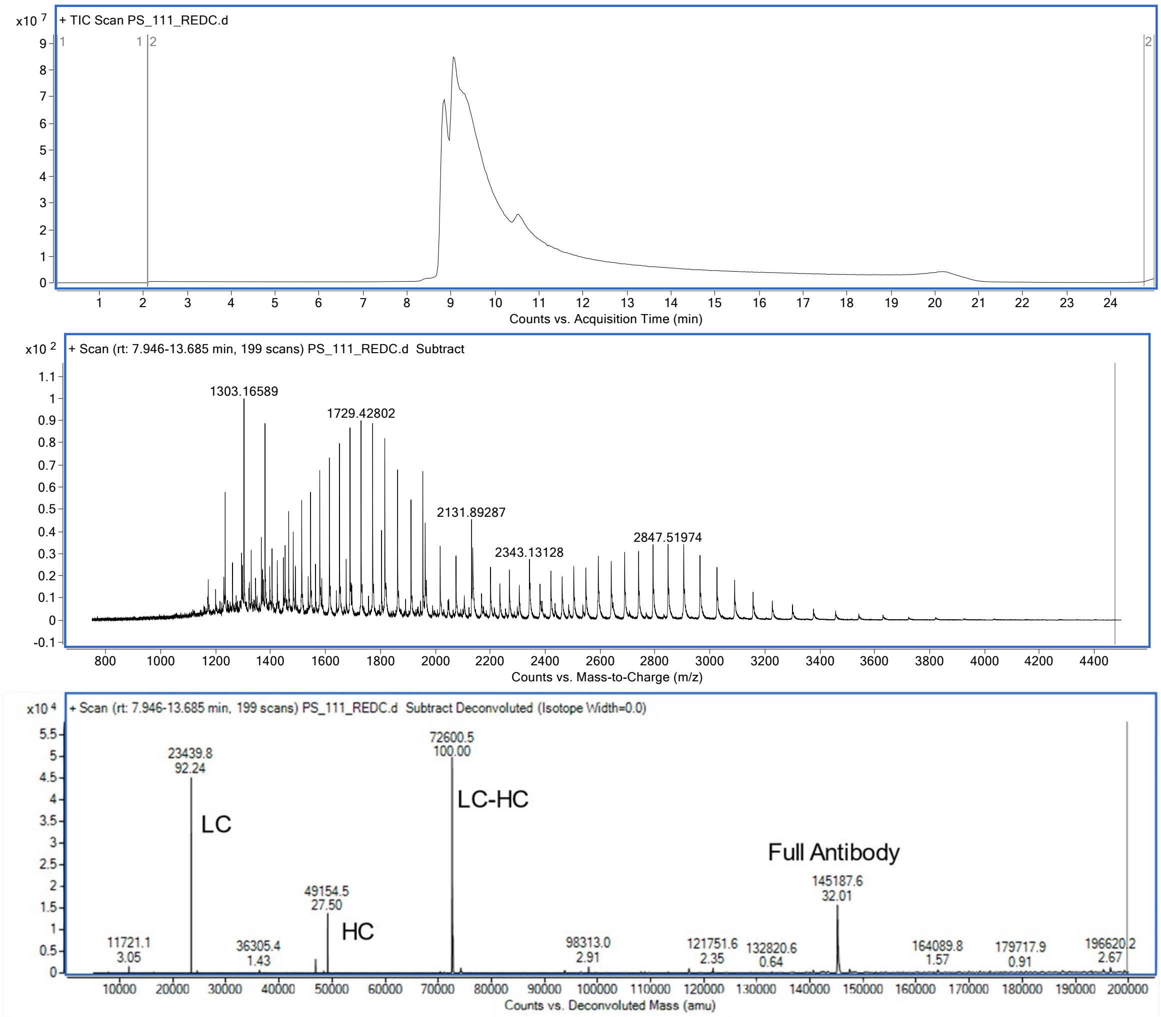


This sample was reduced and then left to re-oxidize over 5 h to showcase the different fragments derived from trastuzumab that can be expected – LC, HC, LC-HC (half-antibody), full antibody.

As trastuzumab is LC-HC-HC-LC, the ratio of LC to HC is expected to be 1:1. The spectrum shows that the apparent ratio of LC to HC is in fact 1:0.3. This suggests that ionization of HC is inferior to that of LC and is thus underrepresented in the MS spectrum.

To further compound this issue, HC has 3 available disulfides and incomplete modification of these would result in multiple species. Thus, the intensity of the HC peak may be further reduced by 'dilution' into 4 different subspecies (HC, HC-mod, HC-(mod $)_2$, HC-$(mod)_3$) leading to the mass envelopes merging with the background, making the deconvolution algorithm unable to resolve them.

As LC has only one cysteine and ionizes much better, it is a better candidate for evaluation of reaction efficiency.

**Figure S23.** Identification and analysis of trastuzumab bioconjugation with **3** dye, at pH = 8.0

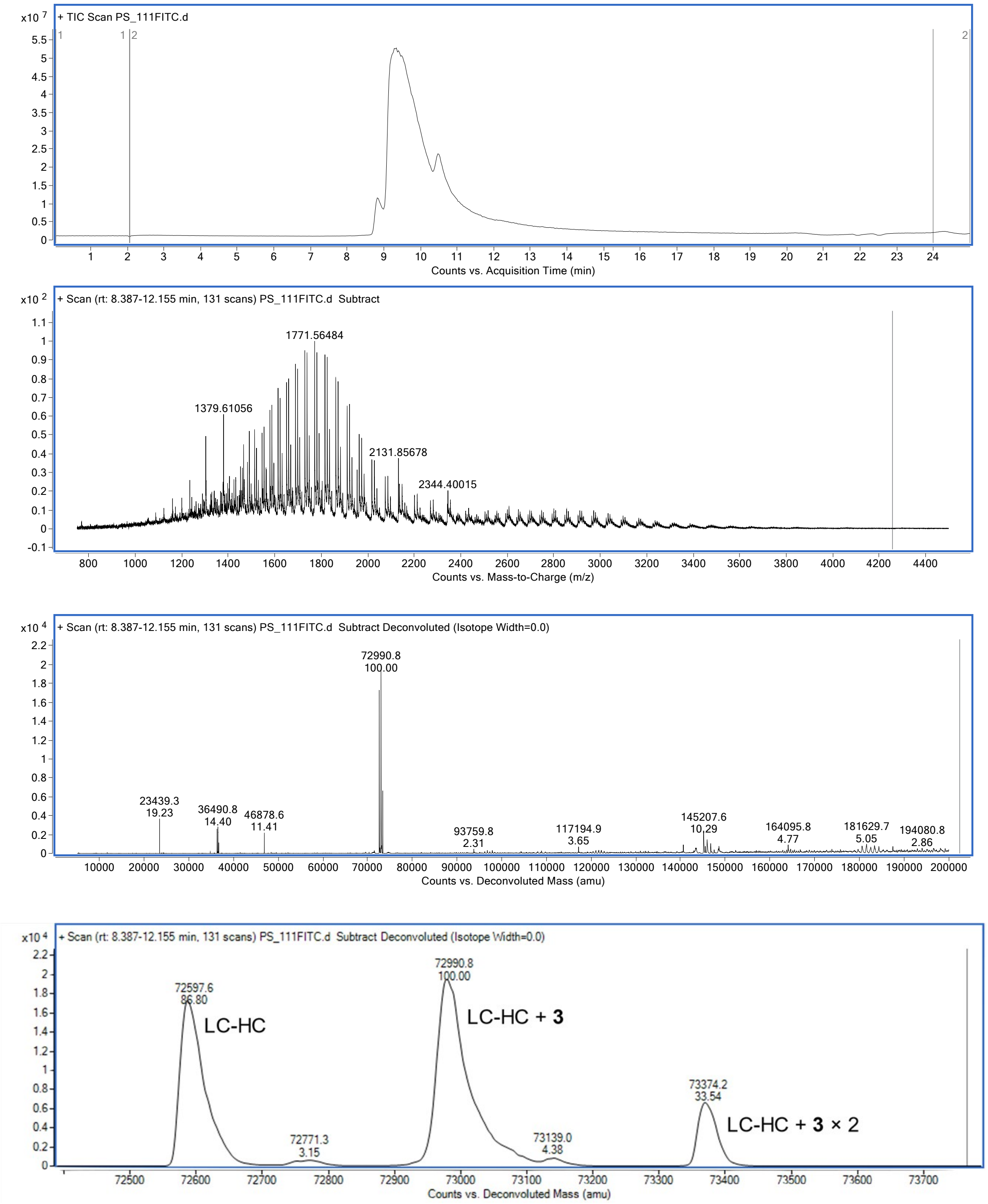


Efficiency of reaction was appraised on the most abundant ionic species: HC-LC (half-antibody). LC-HC being the main species in of itself suggests inefficient labelling as at least one HC and LC cysteine must have been unmodified for the disulfide bridge to re-form and produce LC-HC. Furthermore, it is shown that from the total of 4 cysteines represented in the LC-HC fraction of the sample (1 on LC and 3 on HC) in total only 18.9% was modified with **3** while 81.9% remained unmodified (based on abundance of ionic species).

**Figure S24.** Identification and analysis of trastuzumab bioconjugation with **4** dye, at pH = 8.0

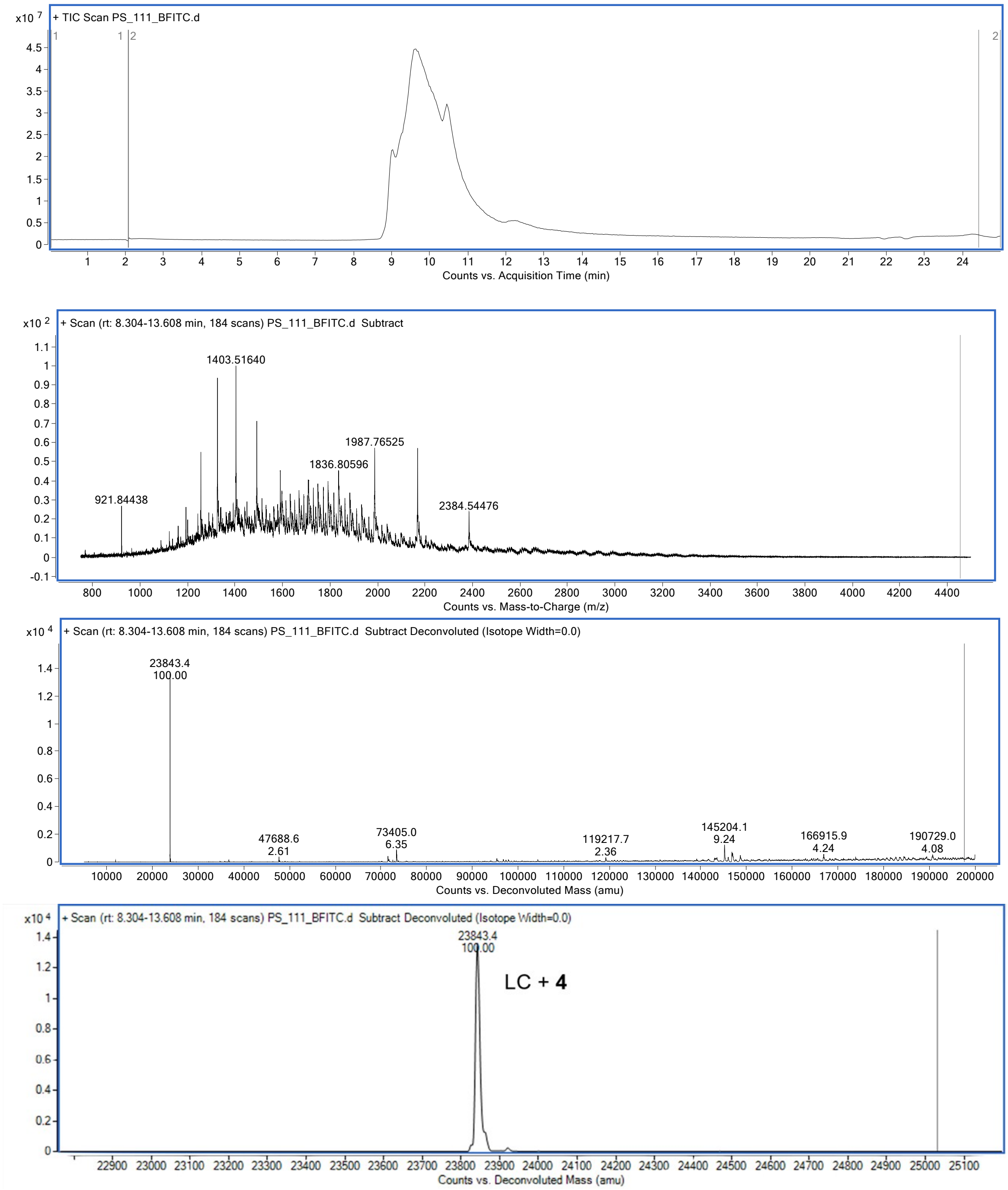


Efficiency of reaction was appraised on the most abundant ionic species: LC. LC being the main ionic species suggests that most cysteines must have been modified as unmodified species would have undergone re-oxidation resulting in larger ionic species (e.g. LC-HC, full antibody). 100% of LC species was modified with **4**.

While the reaction with **4** seems to have decreased the ionization capability of HC to an extent where it does not show up in our spectra (see figure S20 for a rationale for this), as we know from our Fab studies that **4** efficiently labels Fab HC (Figure S14), we can tentatively infer that HC must have been labelled at least somewhat efficiently, especially as unmodified HC would be expected to appear in the spectra (see Figure S20).

## NMR spectra of synthesized compounds

2-(6-hydroxy-3-oxo-3*H*-xanthen-9-yl)-4/5-methylbenzoic acid (**7**)

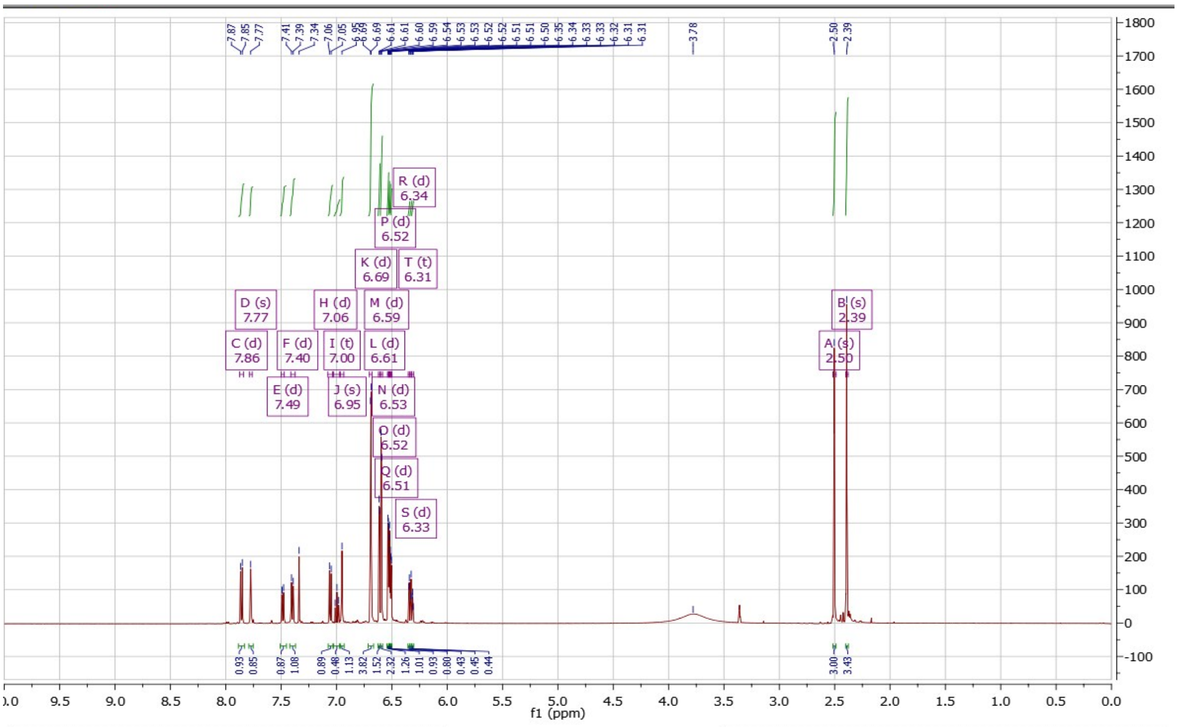


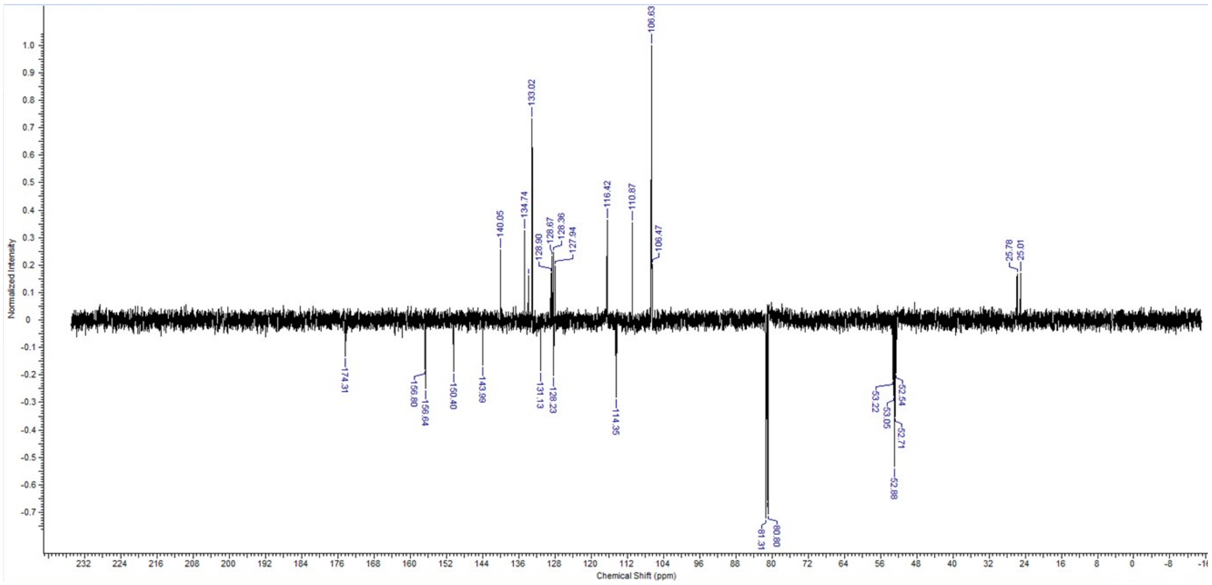

4/5-methyl-3-oxo-3H-spiro[isobenzofuran-1,9'-xanthene]-3',6'-diyl diacetate (**8**)

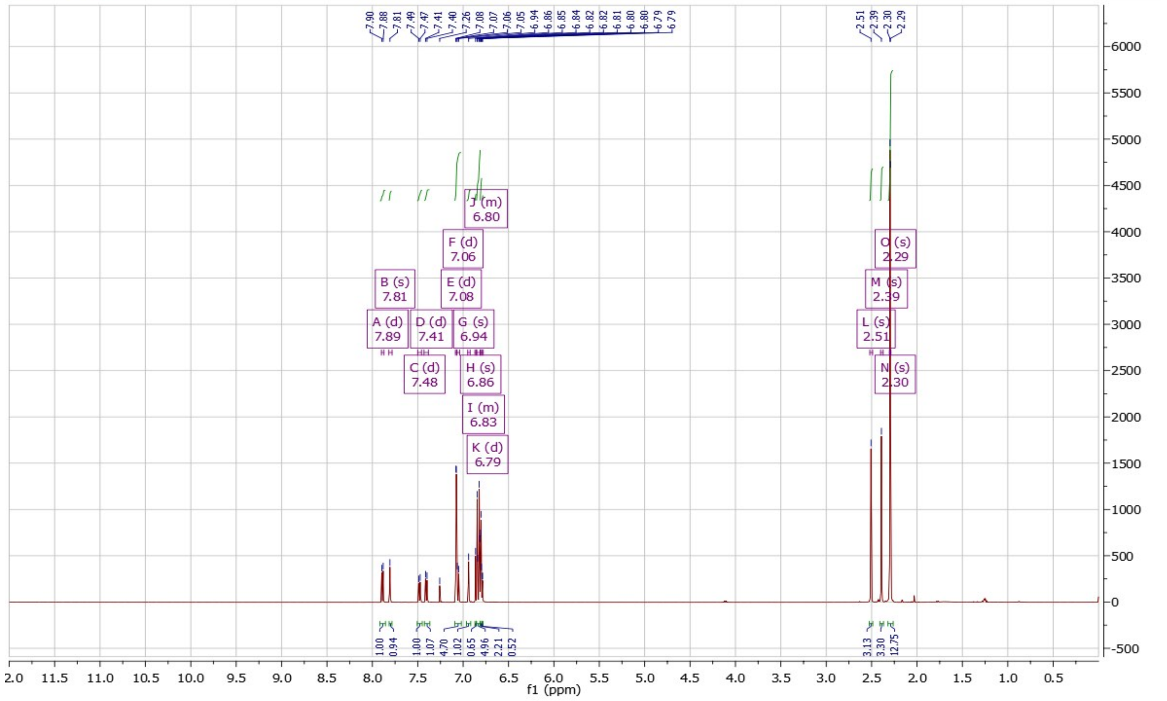

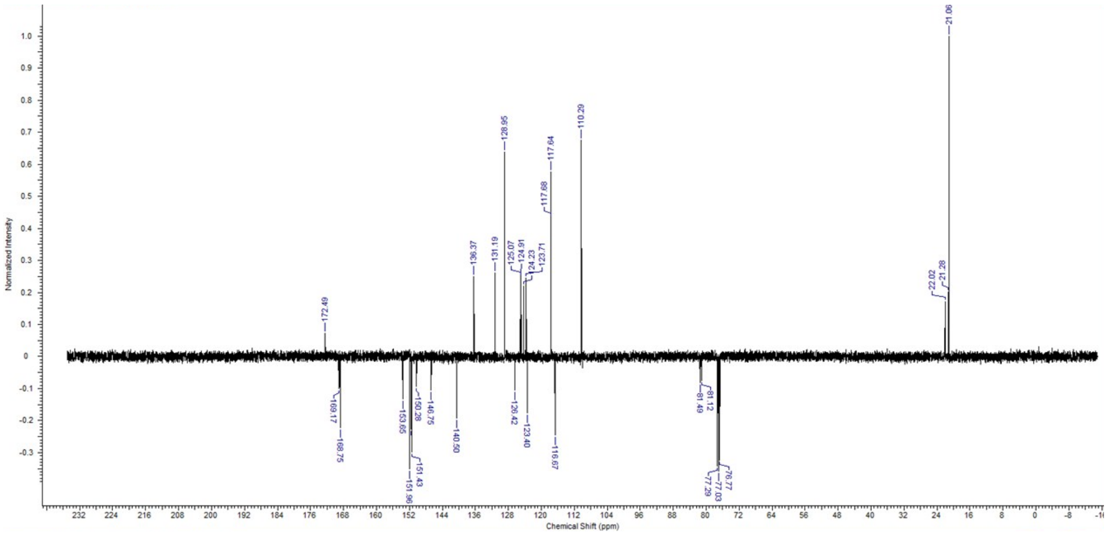

4/5-(bromomethyl)-3-oxo-3H-spiro[isobenzofuran-1,9'-xanthene]-3',6'-diyl diacetate (**9**)

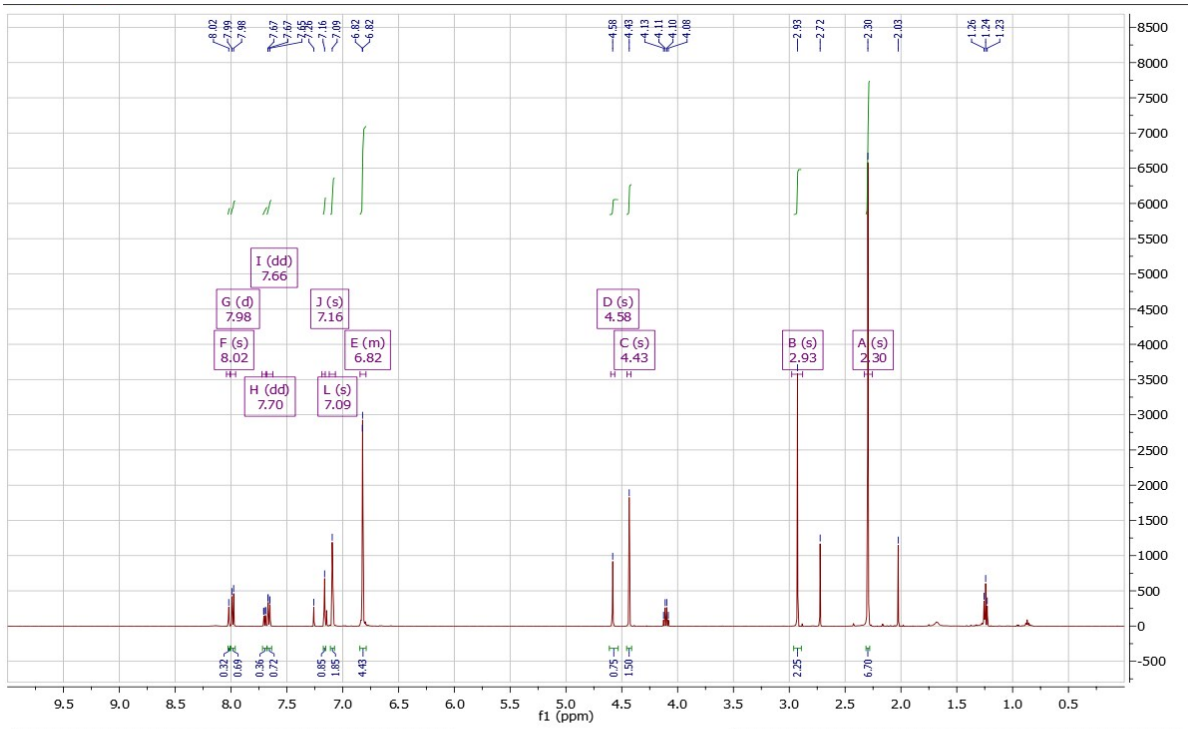

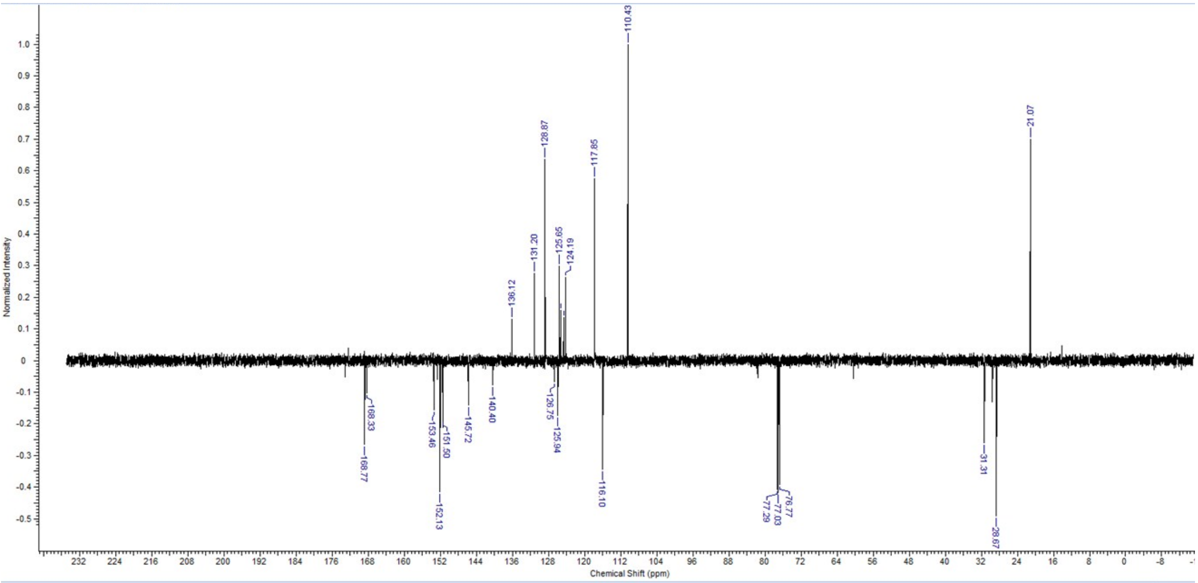

4/5-(aminomethyl)-2-(6-hydroxy-3-oxo-3H-xanthen-9-yl)benzoic acid (**10**)

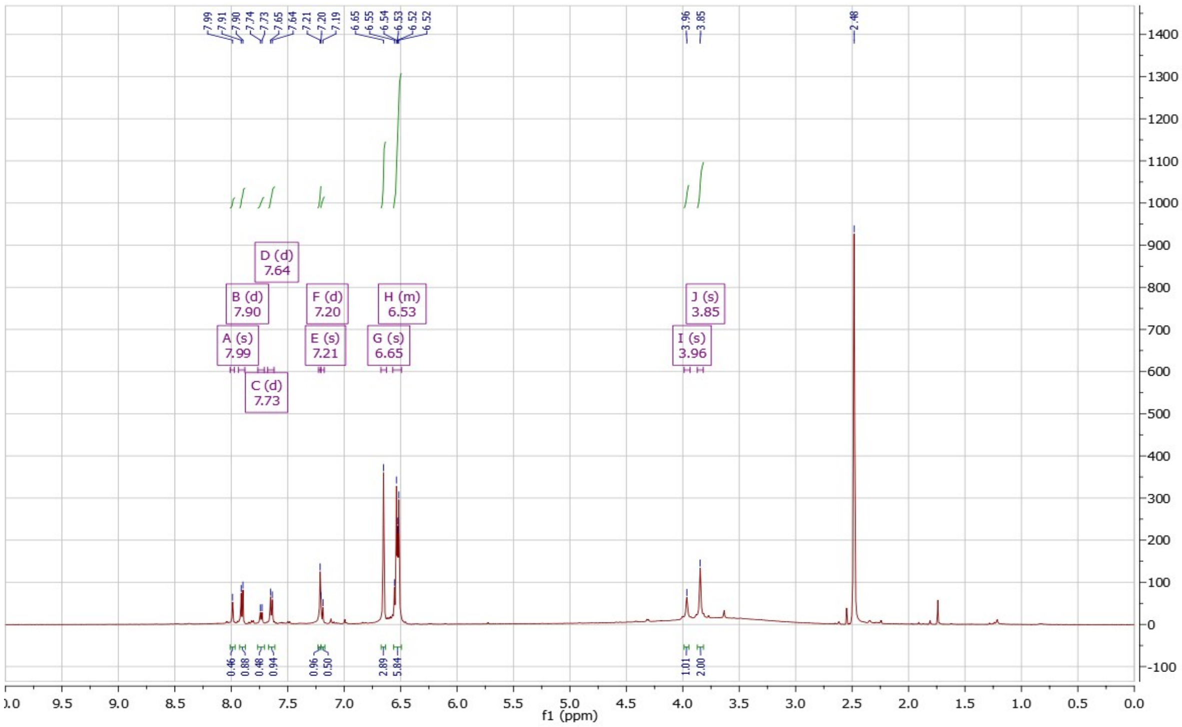

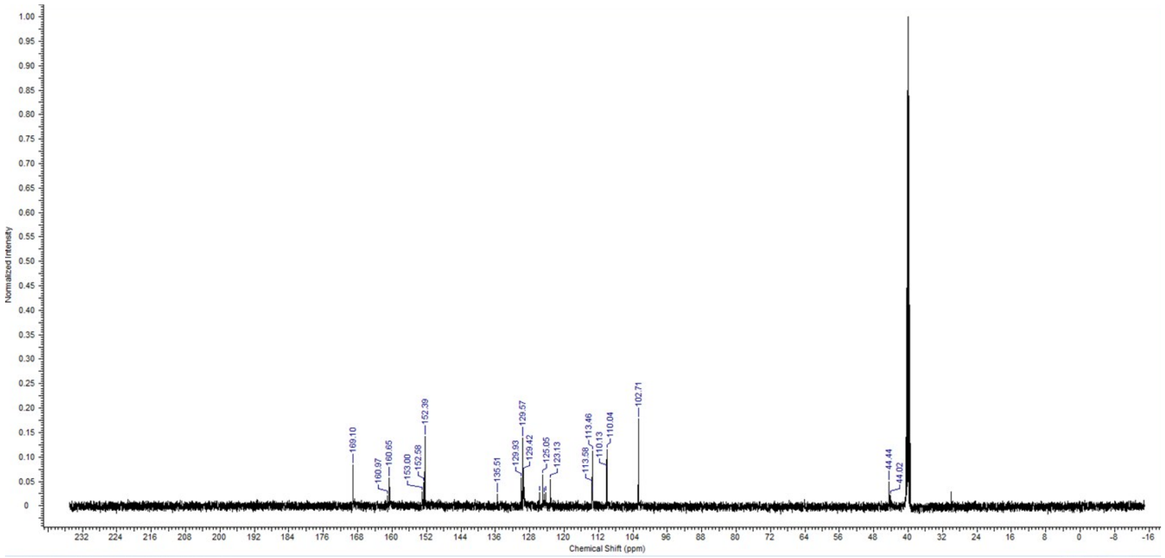

2-(6-hydroxy-3-oxo-3*H*-xanthen-9-yl)-5-(isothiocyanatomethyl)benzoic acid (**4**)

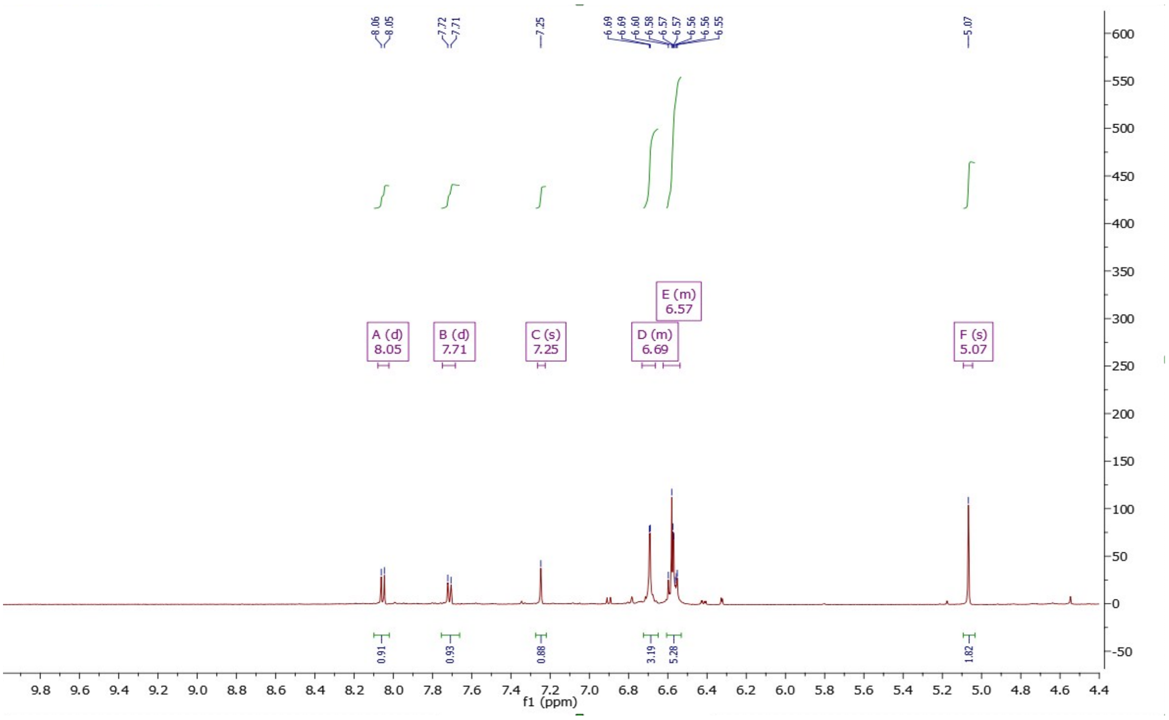


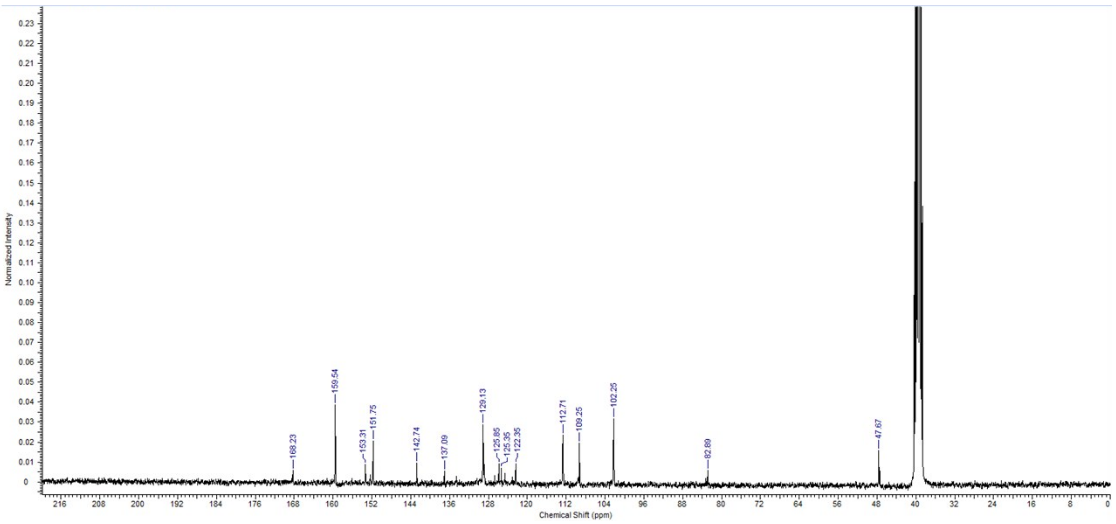

Supplement: RA-010-D0RA02934C-s001 [file RA-010-D0RA02934C-s001.pdf]
